# RUNX2 Activation in Fibro/Adipogenic Progenitors Promotes Muscle Fibrosis in Muscular Dystrophy

**DOI:** 10.1002/advs.202510850

**Published:** 2025-12-22

**Authors:** Pengkai Wu, Zehua Zhang, Kai Zheng, Ziqi Zhu, Yong Zhu, Abdukahar Kiram, Lei Zhao, Huihui Chen, Zhu Xu, Xihua Li, Beicheng Sun, Zhijian Li, Dengqiu Xu

**Affiliations:** ^1^ Department of Hepatobiliary Surgery The First Affiliated Hospital of Anhui Medical University Hefei Anhui China; ^2^ MOE Innovation Center for Basic Research in Tumor Immunotherapy Hefei Anhui China; ^3^ Anhui Province Key Laboratory of Tumor Immune Microenvironment and Immunotherapy Hefei Anhui China; ^4^ The Affiliated Mental Health Center of Jiangnan University Wuxi Central Rehabilitation Hospital Wuxi Jiangsu China; ^5^ Division of Spine Surgery, Department of Orthopedic Surgery, Nanjing Drum Tower Hospital, Affiliated Hospital of Medical School Nanjing University Nanjing Jiangsu China; ^6^ Department of Neurology Children's Hospital of Fudan University Shanghai China; ^7^ Uyghur Medical Hospital of Xinjiang Uyghur Autonomous Region Ürümqi China; ^8^ Xinjiang Key Laboratory of Evidence‐Based and Translation Hospital Preparation of Traditional Chinese Medicine Ürümqi China

**Keywords:** FAPs, fibrosis, macrophages, muscular dystrophy, RUNX2

## Abstract

Clinical evidence indicates concurrent muscle inflammation and fibrosis in muscular dystrophies (MDs); however, the molecular mechanisms underlying inflammation‐mediated fibrosis in skeletal muscle remain inadequately understood. This study revealed a molecular link between macrophages and fibro‐adipogenic progenitors (FAPs) in both human subjects and mice via the transforming growth factor‐beta (TGF‐β)‐RUNX family transcription factor‐2 (RUNX2) axis. *RUNX2* mRNA levels correlated positively with both the expression of fibrotic genes and the fibrosis area of MD patients. We demonstrated that specific ablation of RUNX2 in FAPs alleviated muscle fibrosis in an animal model of MD. Mechanistically, injured myofibers activated the transcription of chemokine genes, enhancing macrophage recruitment and the release of TGF‐β, which subsequently triggered RUNX2‐mediated transcription of fibrogenic genes in FAPs, promoting muscle fibrosis. Additionally, we demonstrated that CADD522, a RUNX2 inhibitor, protects against muscle fibrosis in both dystrophic and denervated mice. Importantly, the anti‐inflammatory drug prednisolone alleviated muscle fibrosis in MD patients by inhibiting inflammatory cytokine‐mediated RUNX2 activation. Collectively, our findings indicated that the TGF‐β‐RUNX2 axis is a viable target for alleviating muscle fibrosis and related diseases, highlighting potential future research directions.

## Graphical abstract

This study revealed a novel role of the chemokine‐TGF‐β1‐RUNX2 axis in determining the fate of FAP differentiation and modulating muscle fibrosis in patients and mice with muscular dystrophies.

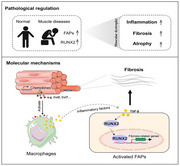

## 1 Introduction

Muscular dystrophies (MDs) are a group of genetic disorders characterized by progressive muscle weakness and wasting, primarily affecting skeletal muscles essential for movement. The main types of MDs include Duchenne muscular dystrophy (DMD), with an estimated incidence of 1 in 3500–5000 male births, and limb‐girdle muscular dystrophy (LGMD), which has an incidence of approximately 1 in 5500 births [1, 2, 3]. These conditions have distinct genetic origins and clinical presentations [4, 5]. DMD arises from mutations in the *DMD* gene, impacting the expression of dystrophin, a crucial muscle structural protein, while LGMD results from mutations in genes encoding dysferlin (Dysf) and sarcoglycans [3, 5]. Current treatments provide symptomatic relief and slow disease progression, but do not offer a cure. Although prednisone and deflazacort have been widely used, they cannot halt muscle function decline in most patients. Research into gene therapies, CRISPR‐Cas9, and other innovative approaches holds promise for more effective future treatments; however, significant challenges remain in achieving widespread adoption and efficacy.

MD has a complex pathophysiology and involves several biological processes, including compromised membrane integrity, dysregulated calcium homeostasis, chronic inflammation, fibrosis, and impaired tissue remodelling [6, 7, 8]. Abnormal dystrophin‐related protein expression disrupts muscle structure, leading to severe tissue degradation exacerbated by persistent inflammation, fibrosis, and necrosis [9, 10, 11]. Continuous inflammation not only damages muscle tissue further but also fosters fibrotic tissue development, worsening muscle function [12, 13]. Transforming growth factor‐beta (TGF‐β) is a primary cytokine that plays a critical role in fibrosis by stimulating fibroblast differentiation into myofibroblasts, which produce large quantities of collagen [14]. While muscle chronic inflammation is recognized as a key factor promoting fibrosis [13, 15], the molecular mechanisms linking inflammation to fibrosis in MD are still not fully understood. Muscle fibrosis treatment remains a significant global challenge, with effective therapies still being limited.

Studies have emphasized the pivotal roles of cellular communication and the muscle microenvironment in maintaining muscle function and health [16, 17, 18]. This influence is intricately connected to the dynamic interplay between fibro‐adipogenic progenitors (FAPs) and their surrounding muscle niche [19, 20, 21, 22, 23]. FAPs, which are nonmyogenic progenitor cells that express platelet‐derived growth factor receptor‐alpha (PDGFRα), reside within muscle tissue [21, 24, 25, 26]. Under normal conditions, FAPs activate and proliferate myoblasts, supporting muscle development and maintenance [22, 27, 28]. However, in MD and during chronic muscle damage, FAPs differentiate into collagen‐producing fibroblasts and adipocytes, leading to muscle fibrosis and fatty infiltration [29, 30, 31]. FAP fate is heavily influenced by the microenvironment, where macrophage‐derived cytokines affect FAP behaviour. Macrophages regulate FAP survival and differentiation by expressing factors such as tumour necrosis factor (TNF) and TGF‐β1, highlighting their essential role in the repair process [30]. Despite this, the mechanisms governing macrophage–FAP interactions within the muscle niche require further clarification.

RUNX family transcription factor 2 (RUNX2) has been implicated in the regulation of mesenchymal cell proliferation in sutures and osteoblast progenitors in both humans and mice [32, 33]. RUNX2 plays a vital role in balancing osteoblast differentiation and bone formation [34]. While it promotes the differentiation of mesenchymal stem cells into osteoblasts in bone, it also drives the differentiation of fibroblasts into myofibroblasts in pulmonary tissue [35]. Although RUNX2's significance in vertebral bone formation and skeletal development in mice is well‐documented [33, 36]. Its role in modulating the muscle microenvironment, particularly in the context of muscular diseases, remains largely unexplored.

In this study, we investigated the role of RUNX2 in inflammation‐mediated muscle fibrosis in MD patients and mouse models, focusing on the macrophage–FAP crosstalk that influences FAP fate. We observed increased RUNX2 expression in the skeletal muscles of dystrophic patients and mice compared to healthy controls. In an animal model of MD, specific ablation of RUNX2 in FAPs significantly alleviated muscle fibrosis. Mechanistically, our findings suggested that damaged myofibers in MDs, including DMD and LGMD, enhance macrophage recruitment and inflammatory infiltration into muscle tissue. Furthermore, inflammatory cytokine‐induced RUNX2 activation drives the transcription of profibrotic genes, exacerbating muscle fibrosis. Collectively, our results revealed a previously unrecognized regulatory pathway involving RUNX2‐mediated interactions between macrophages and FAPs, which play a crucial role in modulating muscle fibrosis in MD. Additionally, we demonstrated that CADD522, a RUNX2 inhibitor, protects against muscle fibrosis in both dystrophic and denervated mice. These findings suggested that targeting FAPs through RUNX2 modulation may offer a novel therapeutic approach for mitigating muscle fibrosis and associated conditions, thereby opening new avenues for future research.

## 2 Results

### 2.1 Muscle Fibrosis Is Linked to Chemokine‐mediated Inflammatory Responses in MD

**FIGURE 1:**
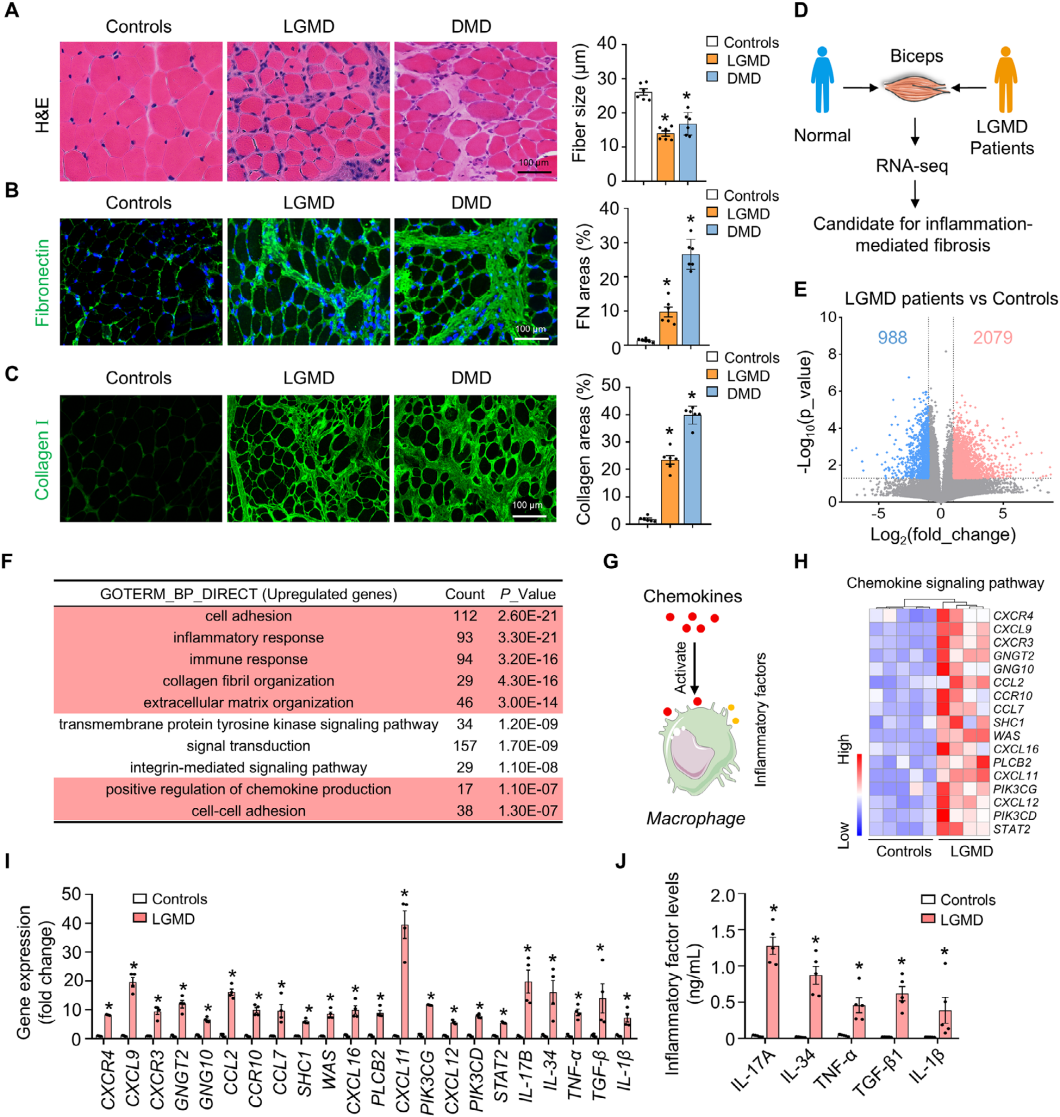
Muscle inflammation‐mediated fibrosis was observed in muscular dystrophy. (A–C) Biceps muscles from male limb‐girdle muscular dystrophy (LGMD) patients, Duchenne muscular dystrophy (DMD) patients, and normal controls were analyzed. *n* = 6 per group. (A: left) HE staining images of skeletal muscle sections from normal controls, LGMD patients, and DMD patients. The scale bar represents 100 µm. (A: right) The mean fiber size was measured by ImageJ. *n* = 6 per group. (B: left) Confocal images showing fibronectin (green) immunostaining in skeletal muscle sections from the indicated groups. The scale bar represents 100 µm. (B: right) Analysis of the fibronectin signal intensity via ImageJ. *n* = 6 per group. (C: left) Confocal images showing collagen I (green) immunostaining in skeletal muscle sections from the indicated groups. The scale bar represents 100 µm. *n* = 6 per group. (C: right) Measurement of the collagen I signal intensity following collagen I immunostaining. *n* = 6 per group. (D to I) Biceps muscles from male LGMD patients and normal controls were analyzed. (D) Schematic of the RNA‐seq experiment. (E) Volcano plot showing fold changes in expression versus *p* values from the RNA‐seq data for the biceps muscles of LGMD patients and controls. Upregulated genes are indicated by red dots; downregulated genes are indicated by blue dots. Male LGMD patients, *n* = 4; normal males, *n* = 5. (F) Biological processes (BPs) enriched in the 2079 upregulated genes in the biceps muscles of LGMD patients according to GO enrichment analysis. (G) Schematic showing how chemokines trigger immune activation and the secretion of inflammatory cytokines within skeletal muscle. (H) Heatmaps showing the RNA‐seq data for chemokine signaling pathway‐related genes found to be upregulated in the biceps muscles of LGMD patients. (I) Analysis of the expression of genes (RT‒qPCR) related to the chemokine signaling pathway and inflammatory response pathway in the triceps muscles of LGMD patients and control subjects. Male LGMD patients, *n* = 4; normal males, *n* = 5. (J) Levels of serum inflammatory markers in LGMD patients versus normal controls by using human IL‐17, IL‐34, TNF‐α, TGF‐β1 and IL‐β ELISA kits. Male LGMD patients. *n* = 5; normal males, *n* = 5. The values represent the means ± SEMs; *p < 0.05 vs. the corresponding controls. *P* values were determined by one‐way ANOVA followed by Fisher's LSD post hoc test (A–C) or two‐tailed unpaired Mann–Whitney test (I and J).

To investigate the relationship between muscle fibrosis and chronic inflammation, we analysed pathological changes in the biceps muscles of 12 MD patients. Given the varying ages of the patients from whom the muscle tissue samples were derived, we examined the pathological changes in LGMD and DMD separately. Haematoxylin and eosin (H&E) staining revealed marked accumulation of inflammatory infiltration and extensive interstitial fibrosis in muscle tissue from both LGMD and DMD patients compared to controls (Figure 1A). Additionally, we observed a significantly reduced average muscle fiber size in LGMD and DMD patients compared to controls (*p* < 0.05) (Figure 1A).

Immunohistochemical analyses further confirmed a significant increase in the protein levels of fibrotic markers, specifically fibronectin and collagen I, within the muscles of both LGMD and DMD patients compared to controls (*p* < 0.05) (Figure 1B,C). Immunoblotting corroborated these findings, demonstrating significantly elevated levels of fibronectin and collagen I in the biceps muscles of both LGMD and DMD patients (*p* < 0.05) (Figure S1A,B). Picrosirius red staining also indicated severe fibrosis in the skeletal muscles of LGMD and DMD patients (Figure S1C). Overall, these results indicated that muscle inflammation and fibrosis are prevalent pathological characteristics of MD patients.

To comprehensively analyse the relationship between muscle fibrosis and chronic inflammation in MD, we performed RNA sequencing (RNA‐seq) analysis of mRNA isolated from the muscle tissues of LGMD patients and controls (Figure 1D). We identified a total of 3067 differentially expressed genes (fold‐change cutoff: 2.0, *p* < 0.05), of which 2079 were upregulated, and 988 were downregulated (Figure 1E). Gene Ontology (GO) enrichment analysis of the upregulated genes revealed significant enrichment in biological processes, such as inflammatory response, cell adhesion, collagen fibril organization, and positive regulation of chemokine production (Figure 1F). RNA‐seq analysis indicated significant upregulation (*p* < 0.05, fold change > 2) of 17 chemokine‐related and 93 inflammatory response‐related genes in the muscles of LGMD patients compared to controls (Figure 1H; Figure S1D). Confirmatory reverse transcription quantitative polymerase chain reaction (RT‐qPCR) demonstrated a dramatic increase in the mRNA levels of chemokine‐, chemokine receptor‐, and inflammatory cytokine‐related genes (Figure 1I). Additionally, enzyme‐linked immunosorbent assay (ELISA) confirmed a marked increase in serum inflammatory cytokine levels in LGMD patients compared to controls (Figure 1J). These data implied that muscle injury is linked to chemokine‐mediated inflammatory responses in LGMD patients.

To explore the role of chemokines in immune activation and inflammatory cytokine secretion in DMD, we analysed the RNA‐seq data from DMD patients and controls. We identified a total of 2039 differentially expressed genes in the muscles of DMD patients, with 1838 being upregulated and 201 downregulated (Figure S2A). GO enrichment analysis of the 1,838 upregulated genes revealed significant enrichment (*p* < 0.05, fold change > 2) in biological processes related to cell adhesion and inflammatory response pathways (Figure S2B). Similar to our findings in LGMD patients, 24 chemokine‐related genes and 52 inflammatory response‐related genes were significantly upregulated (*p* < 0.05, fold change > 2) in the muscles of DMD patients compared to controls (Figure S2C,D). RT‐qPCR confirmed a dramatic increase in the mRNA levels of chemokines, chemokine receptors, and inflammatory cytokine‐related genes (Figure S2E,F) in the muscles of DMD patients. ELISA further validated a significant increase in serum inflammatory cytokine levels in DMD patients compared to controls (Figure S2G). Taken together, these findings suggested that muscle fibrosis is accompanied by chemokine‐mediated inflammatory responses in MD patients.

### 2.2 RUNX2 is a Critical Regulator That Promotes Muscle Fibrosis

**FIGURE 2:**
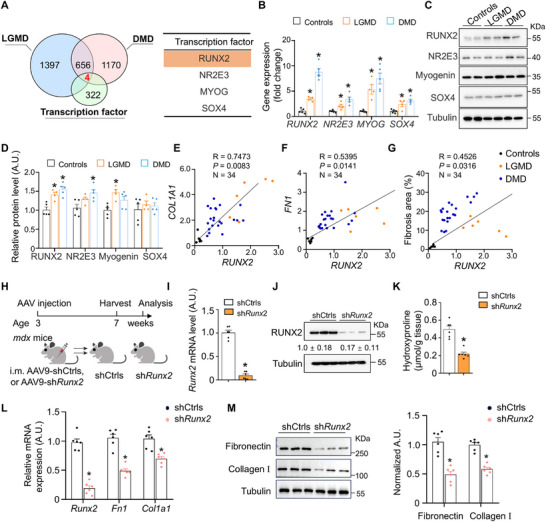
RUNX2 mediates muscle fibrosis in MD. (A) Venn diagrams showing common transcription factor genes that were upregulated in both LGMD patients and DMD patients. (B) RT‒qPCR analysis comparing the mRNA levels of *RUNX2*, *NR2E3*, *MYOG*, and *SOX4* in the biceps muscles of normal controls and LGMD and DMD patients. LGMD patients, n = 4; DMD patients, *n* = 5; normal controls, *n* = 5. (C and D) Western blot analysis demonstrating the protein levels of RUNX2, NR2E3, MYOG, and SOX4 in the triceps muscles of individuals with LGMD, individuals with DMD, and controls. LGMD patients, *n* = 4; DMD patients *n* = 5; normal controls, *n* = 5. (E‒G) Correlation between *RUNX2* gene expression and that of fibrosis‐related genes and the percentage of fibrosis area in Sirius Red staining. *n* = 34 patients. Pearson correlation analysis was used to determine the correlation. (H) Postnatal knockdown of Runx2 in the GAS of *mdx* mice. AAV9‐shCtrls or AAV9‐sh*Runx2* was injected intramuscularly into 3‐week‐old male *mdx* mice. After 4 weeks, all mice were harvested, and GAS muscles were used to analysis. *n* = 6 mice per group. (I) Relative mRNA expression of *Runx2* in the GAS muscles from the indicated mice. *n* = 6 mice per group. (J) Western blots showing RUNX2 protein level in the GAS muscles from the indicated mice. *n* = 6 mice per group. (K) Hydroxyproline levels in DIA were measured with an appropriate kit, and the results were normalized to the relative muscle mass. *n* = 6 mice per group. (L) RT‐qPCR analysis showing the mRNA levels of *Runx2*, *Fn1*, and *Col1a1* in the GAS muscles of the indicated mice. n = 6 mice per group. (M) Western blot analysis showing the protein levels of Fibronectin and collagen I in the GAS muscles of the indicated mice. *n* = 6 mice per group. The values represent the means ± SEMs; *p < 0.05 vs. the corresponding controls. *P* values were determined by two‐tailed unpaired Student's *t*‐test (I and K) and Mann–Whitney test (N, L and M), Pearson's correlation (E, F, and G), and one‐way ANOVA (B and D) followed by Fisher's LSD post hoc test.

Increased collagen deposition and extracellular matrix (ECM) remodeling in fibrotic muscle tissue may be regulated through transcriptional mechanisms. To explore this further, we conducted a detailed analysis of the upregulated genes identified through RNA‐seq. Our analysis revealed that 660 genes were co‐upregulated (*p* < 0.05, fold change > 2) in the muscles of both LGMD and DMD patients. Four of these upregulated genes—*RUNX2*, *NR2E3*, myogenin (*MYOG*), and SRY‐box transcription factor‐4 (*SOX4*)—encode transcription factors (Figure 2A).

RT‐qPCR analysis demonstrated significantly elevated mRNA levels of *RUNX2*, alongside the other three transcription factors, in the biceps muscles of LGMD and DMD patients compared to controls (*p* < 0.05) (Figure 2B). This increase was corroborated by immunoblotting, which revealed elevated protein expression levels for these transcription factors (Figure 2C,D). Similar mRNA and protein expression changes were observed in the gastrocnemius (GAS) muscles of *Dysf*‐knockout (KO) and *mdx* mice, serving as rodent models for LGMD and DMD, respectively (Figure S3A–C). In addition, the levels of *RUNX2* mRNA exhibited a significant positive correlation with fibrosis related‐gene (*COL1A1* and *FN1*) and the percentage of fibrosis area in the skeletal muscle of MD patients (Figure 2E–G). Overall, our results suggested that RUNX2 activation in muscle tissue is involved in the muscle fibrosis associated with MD.

To determine whether muscle fibrosis in MD is dependent on RUNX2 activation, we silenced RUNX2 in *mdx* mice using AAV‐shRNA (Figure 2H). Intramuscular injection of AAV9‐shRNA‐RUNX2 led to efficient knockdown of Runx2 expression, evidenced by markedly reduced Runx2 protein and mRNA levels in skeletal muscle tissues of the AAV9‐shRNA‐RUNX2 group compared to the control group (Figure 2I,J). Additionally, knockdown of Runx2 in muscle significantly alleviated muscle fibrosis, as indicated by decreased hydroxyproline levels in the GAS muscles (Figure 2K). RT‐qPCR and Western blot analyses revealed a reduction in the mRNA and protein levels of fibronectin and collagen I in the GAS muscles of *mdx* mice following AAV9‐shRNA‐RUNX2 treatment (Figure 2L,M). Taken together, these findings demonstrated that RUNX2 regulates muscle fibrosis in MD.

### 2.3 Specific Ablation of RUNX2 in FAPs Alleviated Muscle Fibrosis in mdx Mice

**FIGURE 3:**
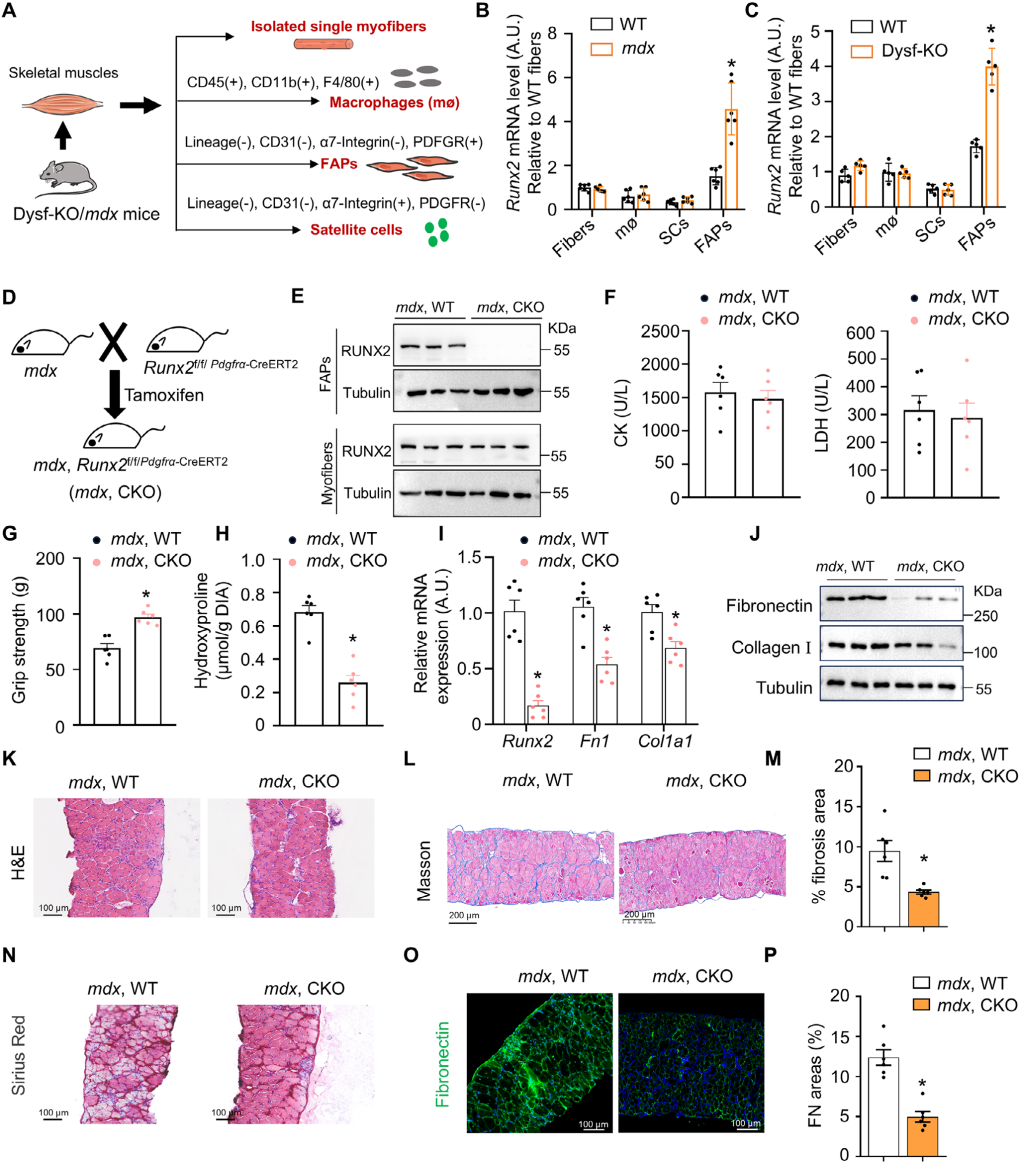
Loss of Runx2 in FAPs alleviates muscle fibrosis in mdx mice. (A) Schematic diagram of the isolation of single fibers, macrophages, FAPs, and muscle satellite cells from the skeletal muscles of 5‐week‐old Dysf‐KO and *mdx* mice. (B) RT‐qPCR analysis of *Runx2* expression in single fibers, macrophage, satellite cells, and FAPs from the skeletal muscles of *mdx* mice and those of controls. *n* = 6 mice per group. (C) RT‒qPCR analysis of *Runx2* expression in single fibers, macrophage, satellite cells, and FAPs from the skeletal muscles of Dysf‐KO mice relative to those from the skeletal muscles of control mice. *n* = 6 mice per group. (D) Diagram illustrating the strategy used to generate *Runx2* ^f/f/PDGFRα–Cre ER^, *mdx* mice. 8‐week‐old mice were administered Tamoxifen daily for 5 consecutive days, and tissue samples were collected for analysis 3 weeks post‐treatment. *n* = 6 mice per group. (E) Western blot analysis showing the protein levels of Runx2 in the primary FAPs and myofibers from the indicated 12‐week‐old male mice. *n* = 3 mice per group. (F) CK and LDH levels were tested with the appropriate kits. *n* = 6 mice per group. (G) Grip strength was measured in the indicated mice at the age of 12 weeks. *n* = 6 mice per group. (H) Hydroxyproline levels in DIA were measured with an appropriate kit, and the results were normalized to the relative muscle mass. *n* = 6 mice per group. (I) RT‐qPCR analysis showing the mRNA levels of *Runx2*, *Fn1*, and *Col1a1* in the DIA muscles of the indicated mice. *n* = 6 mice per group. (J) Western blot analysis showing the protein levels of Fibronectin and collagen I in the DIA muscles of the indicated mice. *n* = 6 mice per group. (K) HE‐stained DIA sections from the indicated mice. The scale bar represents 100 µm. *n* = 6 mice per group. (L) Representative images of Masson‐stained DIA muscles from the indicated male mice. The scale bar represents 200 µm. *n* = 6 mice per group. (M) Quantification of the percentage of fibrosis area via ImageJ. *n* = 6 per group. (N) Representative images of Picrosirius red‐stained DIA muscles from the indicated male mice. The scale bar represents 100 µm. *n* = 6 mice per group. (O) Representative immunofluorescence images of fibronectin (green) and DAPI (blue) in DIA muscles cross sections from the indicated mice. The scale bar represents 100 µm. *n* = 6 mice per group. (P) Analysis of the fibronectin coverage area based on the images in (O). *n* = 6 mice per group. The values represent the means ± SEMs; *p < 0.05 vs. the corresponding controls. *P* values were determined by two‐tailed unpaired Student's *t*‐test (G, H, M, and P) and Mann–Whitney test (B, C, and I).

To further elucidate the mechanism by which RUNX2 mediates fibrosis in dystrophic muscles, we utilized specific in vitro models to isolate single myofibers, macrophages, FAPs, and satellite cells from both *mdx* and *Dysf*‐KO mice (Figure 3A). RT‐qPCR results indicated significantly elevated *Runx2* mRNA levels in the FAPs of *mdx* mice (*p* < 0.05) (Figure 3B) and *Dysf*‐KO mice (Figure 3C) compared to wild‐type (WT) controls, while no differences in *Runx2* mRNA levels were observed in single myofibers, macrophages or satellite cells. We then examined the mRNA levels of the other three identified transcription factors in these specific in vitro models. Notably, *Nr2e3* expression was significantly increased in the satellite cells of both *Dysf*‐KO and *mdx* mice (*p* < 0.05) (Figure S3D–I). Moreover, *MyoG* expression was elevated in the myofibers of *Dysf*‐KO and *mdx* mice, while *Sox4* expression was markedly increased in the myofibers, macrophages, satellite cells, and FAPs of these models (Figure S3D–I). Additionally, RUNX2 was localized in the nuclei of FAPs in both MD patients (Figure S3J) and MD mouse models (Figure S3K), further supporting its role in muscle fibrosis.

To investigate the in vivo role of FAP‐derived RUNX2 in fibrosis, we generated FAP‐specific *Runx2*‐KO mice (referred to as *Runx2* CKO mice) by crossbreeding mice with a conditional null allele for *Runx2* and PDGFR‐α promoter‐driven Cre mice. Subsequently, we created FAP‐specific *Runx2*‐KO *mdx* mice (*mdx* CKO mice) through crossbreeding *Runx2* CKO mice with *mdx* mice (Figure 3D). As anticipated, RUNX2 protein levels were markedly reduced in the FAPs of *mdx* CKO mice compared to *mdx* WT mice, while RUNX2 protein levels remained unchanged in the myofibers (Figure 3E). Interestingly, *mdx* CKO mice showed no differences in CK or LDH levels (Figure 3F), but exhibited significant improvements in grip strength (Figure 3G) compared to *mdx* WT mice. Moreover, *mdx* CKO mice displayed a lower hydroxyproline level in the DIA muscles compared to that in *mdx* WT mice (Figure 3H). RT‐qPCR and Western blot analyses revealed a reduction in the mRNA and protein levels of fibronectin and collagen I in the DIA muscles of *mdx* CKO mice (Figure 3I,J). Histological analysis of DIA muscles from *mdx* CKO mice revealed lower ECM deposition and fibronectin expression compared to that in *mdx* WT mice (Figure 3K–P). Collectively, these results highlighted the contribution of FAP‐derived RUNX2 to muscle fibrosis in MD.

### 2.4 Chemokine‐Mediated Secretion of Inflammatory Cytokines Promoted RUNX2 Activation in FAPs

**FIGURE 4:**
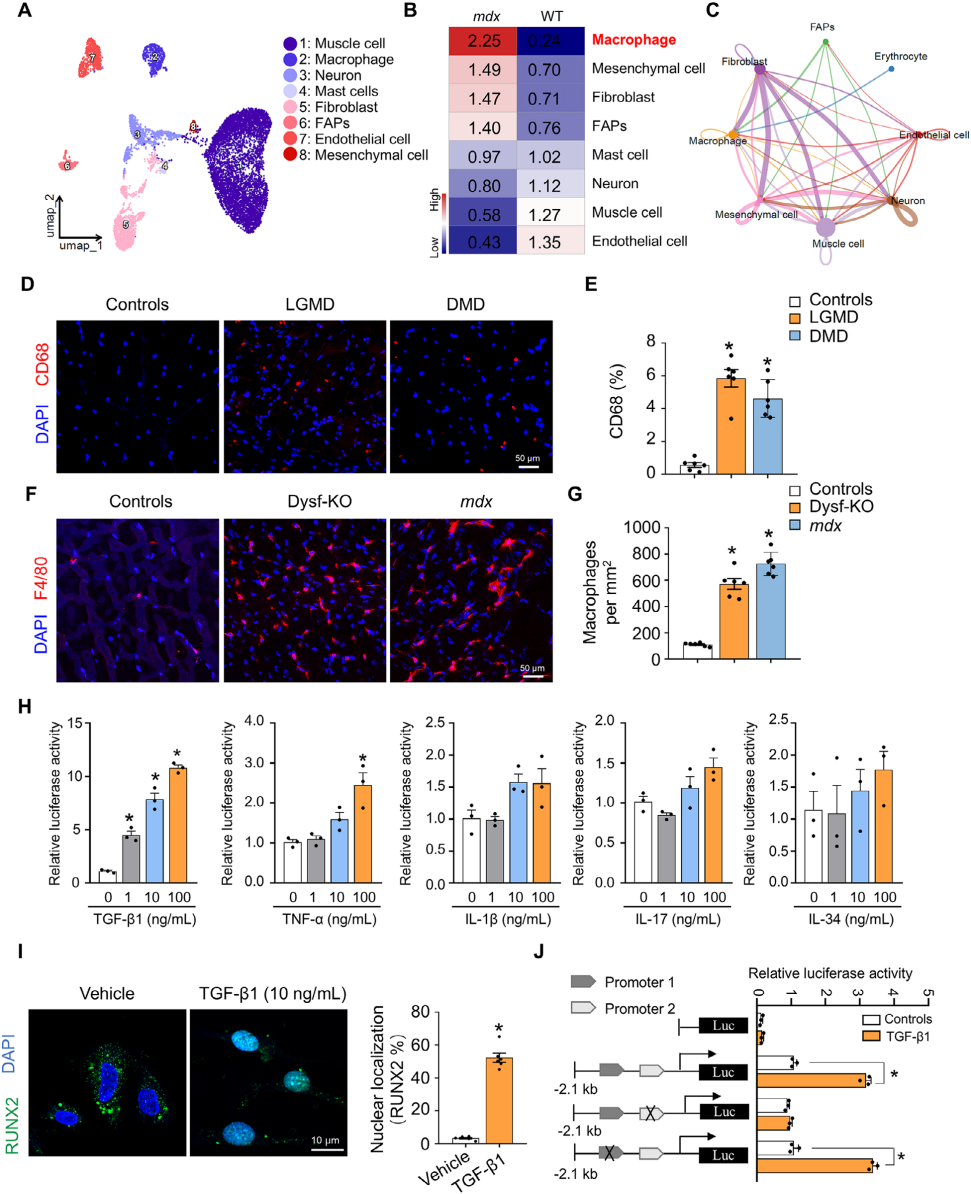
Chemokine‐mediated inflammatory cytokine secretion promotes RUNX2 activation. (A) UMAP analysis of 292,423 sc/snRNA‐seq profiles delineating 8 main skeletal muscle cell populations from single‐nucleus RNA‐seq (snRNA‐seq) data (GSE156498). (B) A heatmap illustrating the cell count for each cell type sequenced, showing an increased presence of macrophages, mesenchymal cells, fibroblasts, and FAPs in the skeletal muscle of *mdx* mice. (C) Cellular network in *mdx* mice; the nodes represent the cell types, and the edges represent the interactions among them. The edge width is proportional to the interaction probability. (D) Confocal images showing CD68 (red) immunostaining in skeletal muscle samples from the specified groups. Scale bar represents 50 µm. (E) Count of CD68‐positive immune cells by ImageJ. *n* = 6 per group. (F) Representative images of F4/80 staining in the GAS muscles of WT, Dysf‐KO, and *mdx* mice. The scale bar represents 50 µm. *n* = 6 per group. (G) Quantification of the number of F4/80‐positive macrophages per mm^2^. *n* = 6 per group. (H) *RUNX2* promoter activity in primary FAPs after treatment with inflammatory cytokines (TGF‐β1, TNF‐α, IL‐1β, IL‐17 or IL‐34). *n* = 3 independent experiments. (I) (left) Representative confocal images of RUNX2 (green) staining in isolated mouse primary FAPs after treatment with or without 10 ng/mL TGF‐β1. The scale bar represents 10 µm. *n* = 3 independent experiments. (right) The quantification of nuclear localization of RUNX2. (J) Generation of different luciferase reporters under the control of the RUNX2 promoter. *RUNX2* promoter activity was tested in primary FAPs after treatment with 10 ng/mL TGF‐β1. *n* = 3 independent experiments. The data are shown as the means ± SEMs. *p < 0.05 vs. the corresponding controls. *P* values were determined by one‐way ANOVA followed by Fisher's LSD post hoc test (E, G, and H) or unpaired two‐tailed Student's *t* test (J).

To further explore the mechanisms underlying muscle inflammation and fibrosis in MD, we examined the interactions between macrophages and FAPs in dystrophic and WT skeletal muscles. Utilizing single‐nucleus RNA‐seq (snRNA‐seq) data (GSE156498) derived from isolated nuclei of skeletal muscle from *mdx* mice in a prior study [37], we employed t‐distributed stochastic neighbour embedding for cell visualization. This analysis revealed eight major cell clusters, identifying muscle cells, stromal cells (including FAPs, fibroblasts, and mesenchymal cells), immune cells (macrophages and mast cells), and endothelial cells as predominant muscle‐resident cell types (Figure 4A). Cell proportion analyses indicated an increased presence of macrophages, mesenchymal cell, fibroblasts and FAPs in *mdx* mice compared to controls (Figure 4B). Furthermore, our data highlighted a significant interplay between macrophages and FAPs, specifically fibroblasts, within dystrophic skeletal muscles (Figure 4C). FAPs have the capacity to differentiate into fibroblasts [30], and RUNX2 is highly expressed in these FAPs (Figure 3B,C). Building on these findings, we hypothesize that macrophage‐derived inflammatory cytokines play a pivotal role as mediators in the crosstalk between macrophages and FAPs.

Histological assessments corroborated the increased recruitment of macrophages in the muscles of both patients and mice with MDs (Figure 4D–G). To ascertain whether macrophage‐secreted cytokines trigger RUNX2 activation in FAPs, we stimulated primary FAPs with five inflammatory cytokines (TGF‐β1, TNF‐α, interleukin [IL]‐1β, IL‐17, and IL‐34) that are commonly elevated in the muscles of LGMD and DMD patients. Subsequently, a luciferase assay was performed to evaluate *RUNX2* promoter activity [8, 14]. Stimulation with TGF‐β1 and TNF‐α resulted in significant increases in *RUNX2* promoter activity, while IL‐1β, IL‐17, and IL‐34 did not elicit notable changes (Figure 4H). Furthermore, TGF‐β1 treatment resulted in increased nuclear localization of RUNX2 in primary FAPs (Figure 4I). We subsequently inserted a 2.1 kb fragment of the mouse *RUNX2* gene promoter region into the PGL3 reporter construct (m*Runx2*.Luc.2.1k). Transfection of m*Runx2*.Luc.2.1k into FAPs followed by TGF‐β1 treatment elicited a marked increase in *RUNX2* promoter activity (Figure 4J). In contrast, deletion of promoter sequences ranging from −1.1 kb to −0.1 kb (promoter 2) abrogated the TGF‐β1‐induced *RUNX2* promoter activity, indicating the presence of a critical cis‐regulatory element responsive to TGF‐β1 within this region (Figure 4J). Collectively, these findings suggest that TGF‐β1 may be the primary cytokine modulating both the promoter activity and nuclear translocation of RUNX2 in muscle FAPs.

Chemokines are integral to macrophage recruitment and the subsequent secretion of inflammatory factors [10, 38]. Specifically, chemokine (C‐C motif) ligand (CCL)2 and CCL7 are key chemokines that facilitate macrophage recruitment and are crucial for mediating muscle inflammation in *mdx* mice [30, 39]. Additionally, our data indicated significant alterations in the mRNA levels of CCL2 and CCL7 within the biceps muscles of both LGMD and DMD patients. Consequently, we investigated the roles of CCL2 and CCL7 in the activation of *RUNX2* and the ensuing development of muscle fibrosis induced by inflammatory cytokines in *mdx* mice. Intramuscular delivery of AAV9‐sh*Ccl2*/*Ccl7* to male *mdx* mice resulted in efficient knockdown of CCL2 and CCL7 in their skeletal muscles relative to the control group (Figure S4A). As anticipated, this intervention led to a marked reduction in inflammatory cytokine‐associated mRNA levels (Figure S4B) and a decrease in macrophage numbers (Figure S4C,D) in the GAS muscles of *mdx* mice, indicating that muscle‐derived chemokines CCL2 and CCL7 are essential for macrophage recruitment and inflammatory cytokine secretion. Furthermore, silencing *Ccl2* and *Ccl7* in the muscle resulted in significant reductions in the protein levels of RUNX2, fibronectin, and collagen I (*p* < 0.05) (Figure S4E). RT‐qPCR results indicated that silencing *Ccl2* and *Ccl7* significantly lowered *Runx2* mRNA levels only in the FAPs of *mdx* mice (*p* < 0.05) (Figure S4F). Histological analyses of *mdx* mouse muscles revealed that silencing *Ccl2* and *Ccl7* ameliorated muscle inflammation and fibrosis (Figure S4G). Collectively, these results highlighted the critical roles of chemokines CCL2 and CCL7 in FAPs RUNX2‐mediated muscle fibrosis.

### 2.5 TGF‐β1 as a Critical Promoter of RUNX2 Activation and Fibrosis‐Related Gene Expression

**FIGURE 5:**
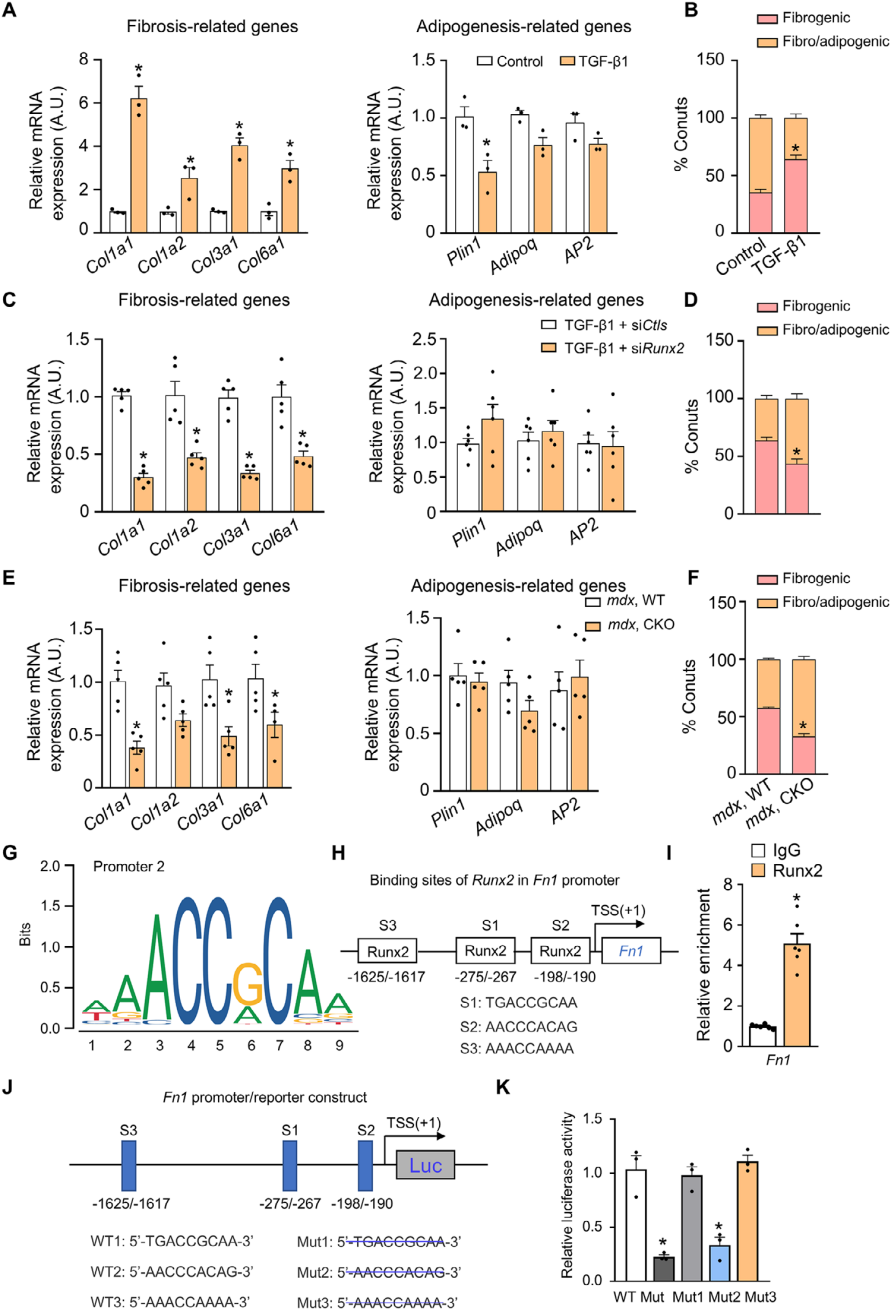
TGF‐β1 promotes RUNX2 activation and fibrosis‐related gene expression in FAPs. (A) RT‐qPCR analysis of the expression of fibrosis‐related genes and adipogenesis‐related genes in isolated FAPs after treatment with 10 ng/mL TGF‐β1. *n* = 3 independent experiments. (B) Quantification of the relative fibrosis‐related genes and adipogenesis‐related genes in isolated FAPs treated with or without TGF‐β1. *n* = 3 independent experiments. (C) RT‐qPCR analysis of the expression of fibrosis‐related genes and adipogenesis‐related genes in isolated FAPs after treatment with 10 ng/mL TGF‐β1 with or without si‐Runx2. *n* = 3 independent experiments. (D) Quantification of the relative levels of fibrosis‐related genes and adipogenesis‐related genes in (C). *n* = 3 independent experiments. (E) RT‐qPCR analysis of the expression of fibrosis‐related genes and adipogenesis‐related genes in the DIA muscles of the indicated 12‐week‐old male mice. *n* = 5 mice per group. (F) Quantification of the relative levels of fibrosis‐related genes and adipogenesis‐related genes in (E). *n* = 5 mice per group. (G) Consensus DNA‐binding motifs of RUNX2 according to the JASPAR database. (H) Predicted binding sites of RUNX2 in the Fn1 promoter according to the JASPAR database. (I) Analysis of Runx2 and IgG occupancy at Fn1 promoter fragments in primary FAPs by ChIP‒qPCR. *n* = 6 independent experiments. (J) Generation of luciferase reporters governed by Fn1 promoter with wildtype or mutant Runx2 binding sites. (K) Results of the dual‐luciferase reporter assay in FAPs co‐transduced with a luciferase reporter driven by the WT or a mutated promoter and a plasmid expressing *RUNX2*. *n* = 3 independent experiments. The data are shown as the means ± SEMs. *p < 0.05 vs. the corresponding controls or vector controls; #*p* < 0.05, TGF‐β1 + si*Runx2* vs. TGF‐β1 + si*Ctrls*. *P* values were determined by one‐way ANOVA followed by Fisher's LSD post hoc test (B, D, F, and K) or unpaired two‐tailed Student's t test (A, C, E, and I).

We investigated how TGF‐β1‐mediated RUNX2 activation promotes muscle fibrosis. Previous studies have shown that FAP differentiation is essential for the progression of muscle fibrosis [21]. Therefore, we examined the mRNA levels of fibrosis‐ and adipogenesis‐associated genes in FAPs treated with 10 ng/mL of TGF‐β1. TGF‐β1 treatment led to significant upregulation of fibrosis‐related genes in FAPs, indicating that TGF‐β1‐mediated RUNX2 activation facilitates FAP fibrogenic differentiation (Figure 5A,B). Subsequently, we investigated the impact of TGF‐β1 on muscle fibrosis using a mouse model in which muscle FAPs were activated by glycerol injection. Administration of 200 ng/kg of TGF‐β1 resulted in a significant increase in protein levels of RUNX2, fibronectin, and collagen I (Figure S5A–D), suggesting an increase in muscle fibrosis. TGF‐β1 treatment specifically elevated the expression of fibrosis‐related genes (*Col1a1*, *Col1a2*, *Col3a1*, and *Col6a1*) in mouse muscle tissue, without affecting the expression of adipogenesis‐related genes (*Plin1*, *Adipoq*, and *AP2*) (Figure S5E). To further determine whether RUNX2 mediates TGF‐β1–induced FAP differentiation, we isolated primary skeletal muscle FAPs from WT and RUNX2 KO mice and treated them with TGF‐β1 (10 ng/mL) or vehicle control. TGF‐β1–induced FAP differentiation was markedly attenuated in RUNX2 KO FAPs, demonstrating that TGF‐β1–mediated FAP differentiation depends on RUNX2 activation (Figure S5F,G). Collectively, these results identify TGF‐β1 as a critical upstream regulator that activates RUNX2 in FAPs to drive fibrogenic differentiation and promote muscle fibrosis.

### 2.6 RUNX2 Orchestrated the Transcription of Fibrosis‐Related Genes

Disruption of FAP homeostasis compromises muscle regeneration, leading to the development of fibrotic and adipogenic lesions in dystrophic muscle [29]. We aimed to elucidate the role of RUNX2 in determining the fate of muscle FAPs by assessing the expression levels of fibrosis‐related and adipogenesis‐related genes. In the presence of TGF‐β1, the application of si‐*Runx2* resulted in a reduction in the expression of fibrosis‐related genes (*Col1a1*, *Col1a2*, *Col3a1*, and *Col6a1*) in FAPs, without altering the expression of adipogenesis‐related genes (*Plin1*, *Adipoq*, and *AP2*) (Figure 5C,D). Similar changes were observed in the diaphragm (DIA) muscles of *mdx* mice with FAP‐specific *Runx2* KO (Figure 5E,F).

To investigate whether RUNX2 directly regulates the identity switching of FAPs, the FAPsChaser (PDGFRα‐ERT‐Cre; mT/mG) system was utilized for pulse–chase lineage‐tracing experiments (Figure S6A). Overexpression of RUNX2 was observed to promote the differentiation of FAPs into collagen‐producing fibroblasts (Figure S6B). These findings underscore the critical role of RUNX2 in regulating the fibrogenic differentiation of muscle FAPs.

Building upon the established role of RUNX2 in liver fibrosis [40, 41], we identified Runx2 as a potential transcriptional regulator of Fibronectin 1 (*Fn1*) through Ingenuity Pathway Analysis. Subsequent analysis using the JASPAR database revealed three potential *Runx2*‐binding motifs within the *Fn1* promoter region, located 190–198, 267–275, and 1,617–1,625 bp upstream of the transcription start site (Figure 5G,H). Chromatin immunoprecipitation (ChIP) coupled with qPCR analysis demonstrated that the *Fn1* promoter exhibited significantly greater binding affinity (∼5.8‐fold increase) for an anti‐Runx2 antibody compared to a nonspecific anti‐IgG antibody in FAPs (*p* < 0.05) (Figure 5I). These results identified *Runx2* as a key transcriptional regulator of *Fn1* and highlighted its role in regulating *Fn1* expression.

We next investigated whether *Runx2* binds to the promoter region of *Fn1* to activate its expression. We mutated the three *Runx2* binding sites in the *Fn1* promoter and individually transfected plasmids containing *Runx2* with either a mutant promoter or the WT promoter of *Fn1* into FAPs. Compared to those expressing *Runx2* with the other mutant promoters, FAPs expressing *Runx2* with a mutation in the second *Fn1* binding site (190–198 bp upstream of the transcription start site) exhibited decreased *Fn1* promoter activity (Figure 5J,K). These data suggested that *Runx2* plays a vital role in regulating *Fn1* expression in FAPs.

Previous studies have also shown that RUNX2 binds to and enhances the activity of the collagen I promoter [40, 41]. Analysis using the JASPAR database revealed three potential Runx2 binding sites within the *Col1a1* promoter region, located 1423–1431 bp upstream, 1952–1960 bp upstream, and 79–87 bp downstream of the transcription initiation site (Figure S7A). ChIP coupled with qPCR analysis revealed higher enrichment efficiency of the *Col1a1* promoter (∼4.7‐fold increase, *p* < 0.05) for an anti‐Runx2 antibody compared to a nonspecific anti‐IgG antibody (Figure S7B). Similar experiments involving the transfection of *Runx2* with mutated binding sites demonstrated that the 1423–1431 bp region of the *Col1a1* promoter is critical for Runx2‐mediated *Col1a1* promoter activation (Figure S7C,D). Collectively, these findings strongly suggested that Runx2 orchestrates the transcription of fibrosis‐related genes, serving as a key transcriptional regulator of the fate of muscle FAPs and contributing to muscle fibrosis.

### 2.7 CADD522, a RUNX2 Inhibitor, Protected Against Muscle Fibrosis in Mice

**FIGURE 6:**
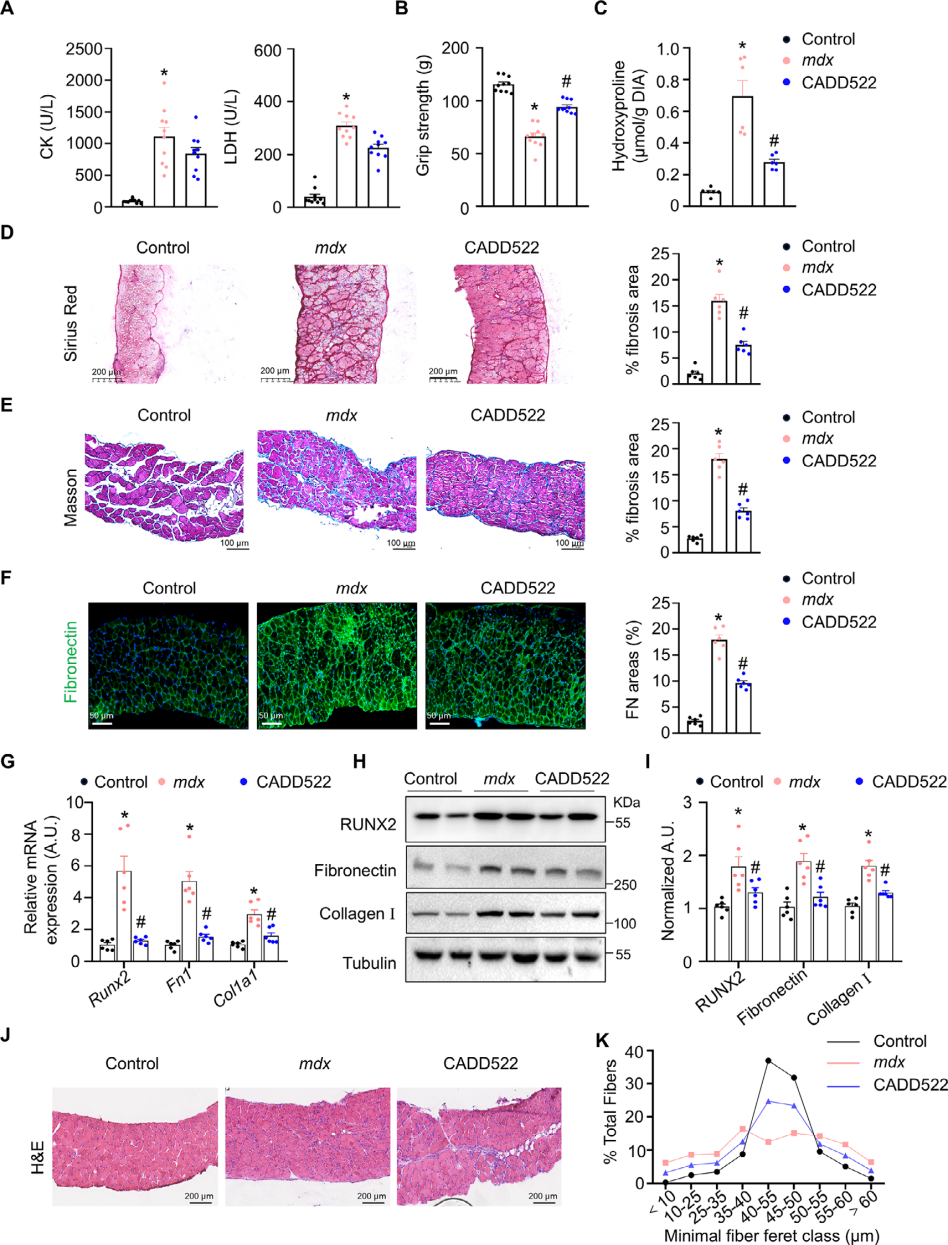
The RUNX2 inhibitor CADD522 protects against muscle fibrosis in *mdx* mice. (A) Serum CK and LDH levels were tested from male *mdx* mice at the age of 12 weeks after treatment with vehicle or CADD522 (10 mg/kg) for 6 weeks with the appropriate kits. *n* = 9 mice per group. (B) Grip strength was measured in indicated mice. *n* = 9 mice per group. (C) Hydroxyproline levels were measured with an appropriate kit, and the results were normalized to the muscle mass. *n* = 6 mice per group. (D) Representative images of Picrosirius red‐stained DIA muscles from the indicated male mice. The scale bar represents 200 µm. *n* = 6 mice per group. (E) Representative images of Masson‐stained DIA muscles from the indicated mice. The scale bar represents 100 µm. *n* = 6 mice per group. (F) Representative immunofluorescence images of fibronectin (green) and DAPI (blue) in DIA muscles cross sections from the indicated mice. The scale bar represents 50 µm. *n* = 6 mice per group. (G) RT‐qPCR analysis showing the mRNA levels of *Runx2*, *Fn1*, and *Col1a1* in the DIA muscles of the indicated 12‐week‐old male mice. *n* = 6 mice per group. (H) Western blot analysis showing the protein levels of RUNX2, fibronectin, and collagen I in the DIA muscles of the indicated 12‐week‐old male mice. *n* = 6 mice per group. (I) RUNX2/Tubulin, fibronectin/Tubulin, and collagen I/Tubulin expression in each group was normalized to that in the control group. *n* = 6 mice per group. (J) Representative images of HE‐stained DIA muscles from control mice and *mdx* mice treated with or without CADD522. The scale bar represents 200 µm. *n* = 6 mice per group. (K) Quantification of the percent frequency distribution of DIA fiber as determined by ImageJ. The data are shown as the means ± SEMs. *p < 0.05 vs. the control. #*p* < 0.05, CADD522 vs. the *mdx* group. *P* values were determined by one‐way ANOVA followed by Fisher's LSD post hoc test.

CADD522 is a well‐characterized inhibitor of RUNX2, disrupting DNA binding through its interaction with the runt box domain protein [32]. We first evaluated the therapeutic potential of CADD522 (10 mg/kg, administered via intraperitoneal injection twice a week) in *mdx* mice, following the protocol established in a previous study [42]. CADD522‐treated *mdx* mice showed no differences in plasma CK or LDH levels (Figure 6A) but exhibited significant improvements in grip strength (Figure 6B). Additionally, CADD522 treatment significantly alleviated muscle fibrosis, as indicated by decreased hydroxyproline levels in the DIA muscles (Figure 6C) and reduced areas positive for Picrosirius red, Masson's trichrome, and fibronectin in the DIA muscles (Figure 6D–F). RT‐qPCR and Western blot analyses revealed a reduction in the mRNA and protein levels of RUNX2, fibronectin, and collagen I in the DIA muscles of *mdx* mice following CADD522 administration (Figure 6G–I). CADD522 treatment effectively mitigated fibrosis and increased myofiber diameter in the DIA muscles, as demonstrated by haematoxylin and eosin staining (Figure 6J,K). Collectively, these findings indicated that CADD522 significantly improves muscle integrity in DMD and exerts its antifibrotic effects through the inhibition of RUNX2.

**FIGURE 7:**
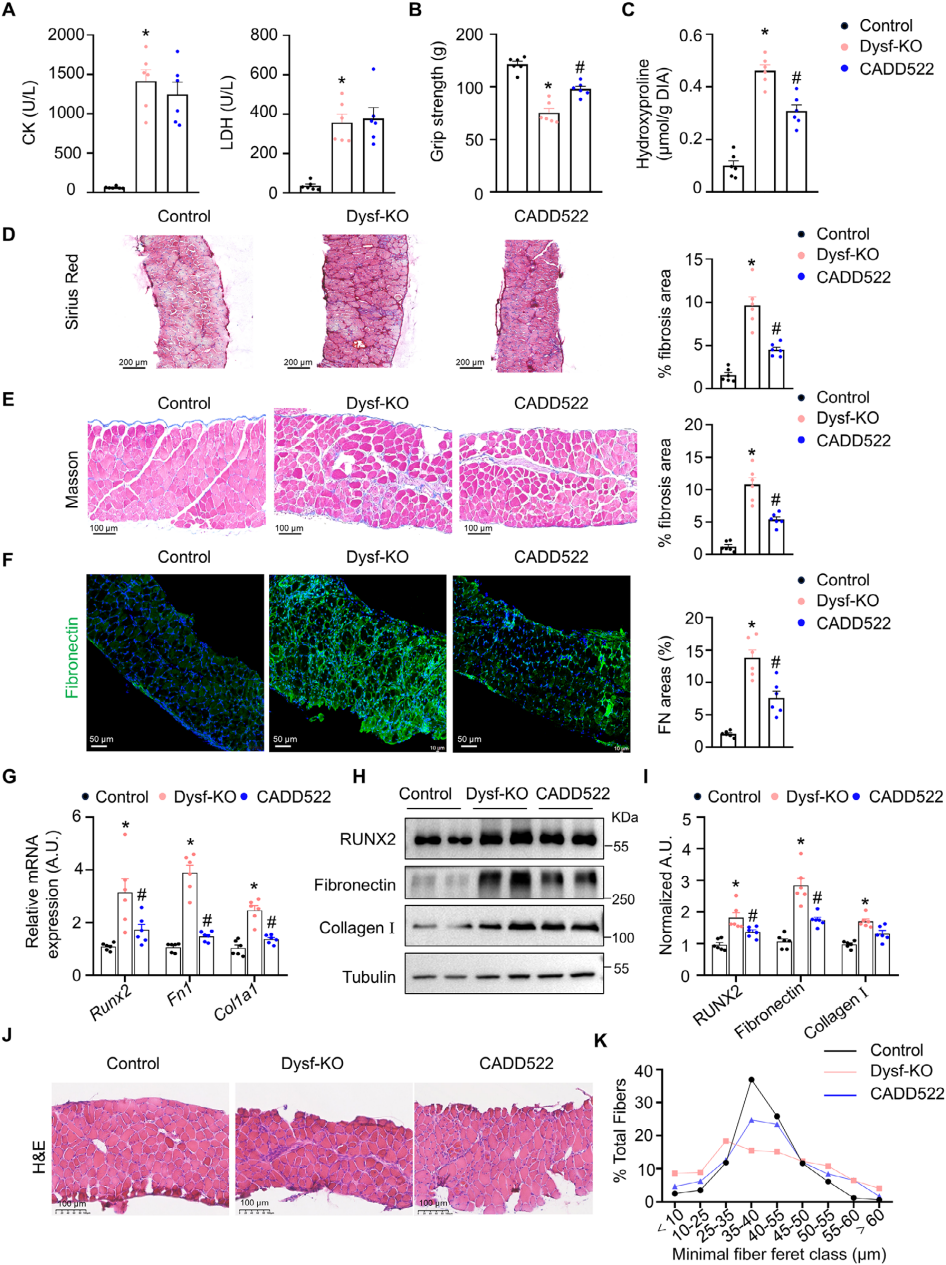
The RUNX2 inhibitor CADD522 protects against muscle fibrosis in Dysf‐KO mice. (A) Serum CK and LDH levels were tested from male Dysf‐KO mice at the age of 6‒8 weeks after treatment with vehicle or CADD522 (10 mg/kg, twice a week) for 4 weeks with the appropriate kits. *n* = 6 mice per group. (B) Grip strength was measured in the indicated mice. *n* = 6 mice per group. (C) Hydroxyproline levels were measured with an appropriate kit, and the results were normalized to the muscle mass. *n* = 6 mice per group. (D) Representative images of Picrosirius red‐stained DIA muscles from the indicated male mice. The scale bar represents 200 µm. *n* = 6 mice per group. (E) Representative images of Masson‐stained DIA muscles from the indicated mice. The scale bar represents 100 µm. *n* = 6 mice per group. (F) Representative immunofluorescence images of fibronectin (green) and DAPI (blue) in DIA muscles cross sections from the indicated mice. The scale bar represents 50 µm. *n* = 6 mice per group. (G) RT‐qPCR analysis showing the mRNA levels of *Runx2*, *Fn1*, and *Col1a1* in the DIA muscles of the indicated 12‐week‐old male mice. *n* = 6 mice per group. (H) Western blot analysis showing the protein levels of Runx2, fibronectin, and collagen I in the DIA muscles of the indicated mice. *n* = 6 mice per group. (I) Runx2/Tubulin, fibronectin/Tubulin, and collagen I/Tubulin expression in each group was normalized to that in the control group. *n* = 6 mice per group. (J) Representative images of HE‐stained DIA muscles from control mice and Dysf‐KO mice treated with or without CADD522. The scale bar represents 100 µm. *n* = 6 mice per group. (K) Quantification of the percent frequency distribution of DIA fiber as determined by ImageJ. The data are shown as the means ± SEMs. *p < 0.05 vs. the control. #*p* < 0.05, CADD522 vs. the Dysf‐KO group. *P* values were determined by one‐way ANOVA followed by Fisher's LSD post hoc test.

The therapeutic potential of CADD522 (10 mg/kg, administered via intraperitoneal injection twice a week) was subsequently assessed in Dysf‐KO mice. Dysf‐KO mice treated with CADD522 (10 mg/kg) exhibited no significant differences in plasma CK or LDH levels (Figure 7A) but showed notable improvements in grip strength (Figure 7B). Muscle fibrosis was significantly alleviated by CADD522 treatment, as evidenced by reduced hydroxyproline levels in the DIA muscles (Figure 7C) and diminished Picrosirius red, Masson's trichrome, and fibronectin‐positive areas (Figure 7D–F). RT‐qPCR and Western blot analyses demonstrated reduced mRNA and protein expression levels of RUNX2, fibronectin, and collagen I in the DIA muscles of Dysf‐KO mice after CADD522 treatment (Figure 7G–I). Fibrosis was effectively mitigated, and muscle fiber diameter was increased by CADD522 treatment in the DIA muscles, as indicated by haematoxylin and eosin staining (Figure 7J,K). Notably, CADD522 (10 mg/kg) exhibited no significant toxicity in the heart, liver, spleen, lungs, or kidneys of dystrophic mice (Figure S8). Collectively, these findings indicate that CADD522 effectively and safely protects against muscle fibrosis in mice.

### 2.8 Prednisolone Inhibited Inflammation‐Mediated RUNX2 Activation and Effectively Reduced Muscle Fibrosis in DMD Patients

**FIGURE 8:**
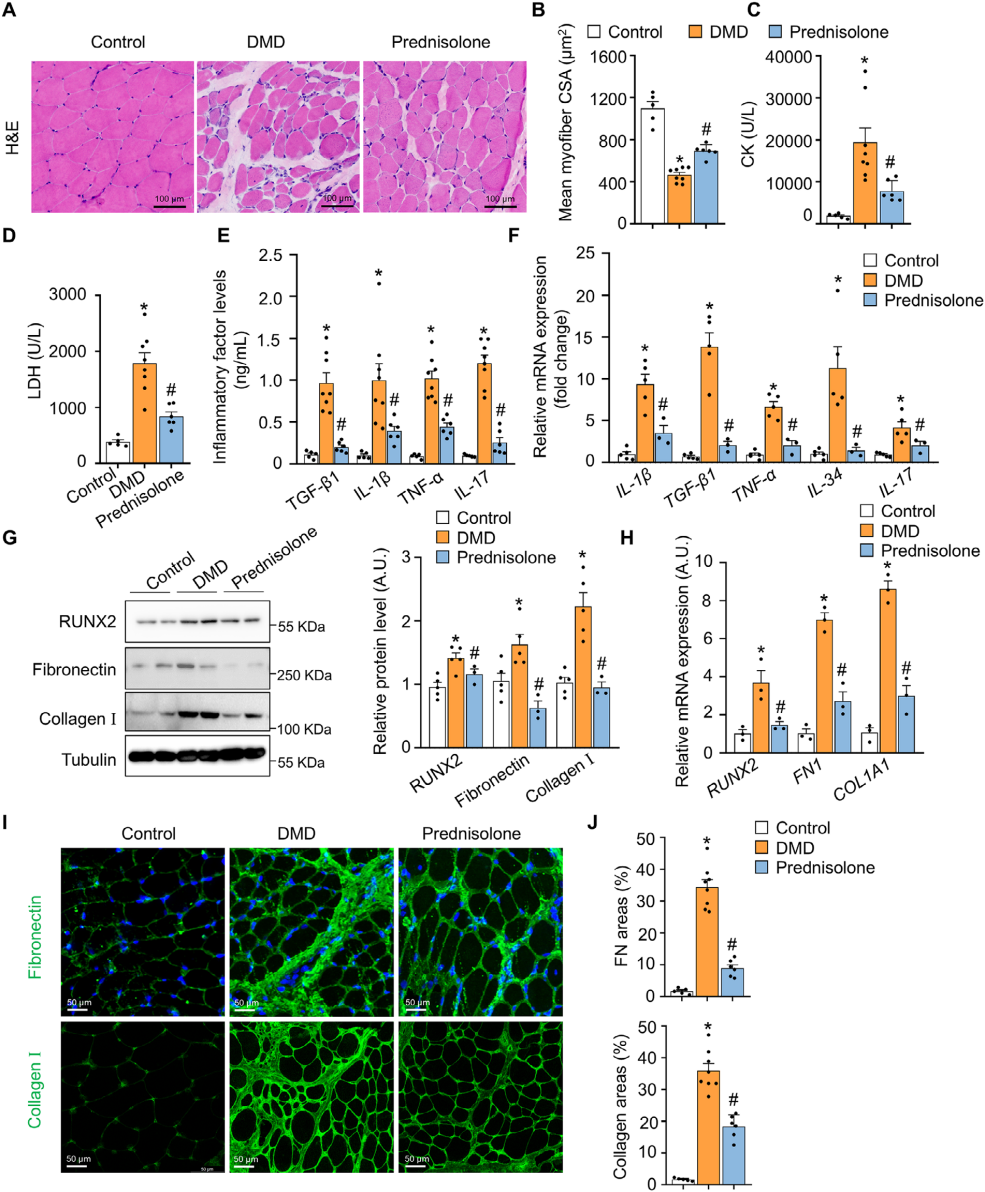
Prednisolone protects against muscle fibrosis and atrophy in patients with DMD. (A) Representative H&E images of samples from the indicated human subjects. The scale bar represents 100 µm. *n* = 5–8 per group. (B) Quantification of muscle CSA from the indicated human subjects. *n* = 5–8 per group. (C) Serum CK level was tested via appropriate kits. *n* = 6‒8 mice per group. (D) Serum LDH level was tested via appropriate kits. *n* = 6‒8 mice per group. (E) Serum TGF‐β1, IL‐1β, TNF‐α, and IL‐17 levels in the indicated human subjects. *n* = 5–8 per group. (F) RT‐qPCR analysis of *IL‐1β*, *TGF‐β1*, *TNF‐α*, *IL‐34*, and *IL‐17* levels in the skeletal muscles of human subjects. *n* = 3–5 per group. (G) Western blot analysis showing the protein levels of Runx2, fibronectin, and collagen I in the skeletal muscles of the indicated subjects. *n* = 3–5 per group. (H) RT‐qPCR analysis of fibrosis‐related genes (*RUNX2*, *FN1*, *COL1A1*) in the skeletal muscles of the indicated subjects. *n* = 3 per group. (I) Confocal images showing fibronectin and collagen I immunostaining in skeletal muscle sections from the indicated subjects. The scale bar represents 50 µm. *n* = 5–8 per group. (J) Measurement of the fibronectin and collagen I signal intensity following immunostaining in (I). *n* = 5–8 per group. The data are shown as the means ± SEMs. *p < 0.05 vs. the corresponding controls; #*p* < 0.05, prednisolone‐treated patients vs. DMD patients. *P* values were determined by one‐way ANOVA followed by Fisher's LSD post hoc test.

Prednisolone, a corticosteroid commonly used in the treatment of DMD, has been shown to reduce inflammation and suppress the immune response, potentially delaying disease progression in DMD patients [43, 44, 45]. To evaluate its efficacy in alleviating inflammation‐related complications and RUNX2 activation‐induced muscle fibrosis, we obtained biceps muscle samples from DMD patients who had undergone prednisolone treatment (0.75 mg/kg) for 6–12 months. Prednisolone treatment significantly increased myofiber CSA (*p* < 0.05) and mitigated pathological changes in the biceps muscle of these patients (Figure 8A,B). Plasma analysis revealed a significant reduction in circulating CK and LDH levels in the prednisolone‐treated group (*p* < 0.05) (Figure 8C,D). These data suggested prednisolone significantly improved whole muscle health. Immunostaining for human cluster of differentiation (CD)68 and CD206 demonstrated that prednisolone markedly reduced inflammatory infiltration in the muscles of DMD patients (Figure S9A,B). ELISA showed significant reductions in plasma levels of inflammatory cytokines, including TGF‐β1, IL‐1β, TNF‐α, and IL‐17 (*p* < 0.05) (Figure 8E), consistent with the reduced mRNA levels of these cytokines in the biceps muscles (Figure 8F). These data demonstrated the anti‐inflammatory effect of prednisolone in DMD patients.

Regarding the relationship between inflammation and fibrosis, we found that decreased levels of inflammatory cytokines were associated with suppressed RUNX2 activation and fibrotic signalling in the skeletal muscles of DMD patients (Figure 8G,H). Immunohistochemical analysis revealed reduced levels of human fibronectin and collagen I following prednisolone treatment (Figure 8I,J). Additionally, we found that prednisolone did not directly affect RUNX2 expression in mouse primary FAPs. Furthermore, prednisolone did not influence the expression of fibrosis‐related genes or adipogenesis‐related genes (*Plin1*, *Adipoq*, and *AP2*) in mouse primary FAPs (Figure S9C,D). These findings suggested that the anti‐fibrotic effect of prednisolone relies on its modulation of immune cell activity and inflammatory cytokine levels.

Interestingly, prednisolone treatment resulted in a marked increase in muscle regeneration capacity in DMD patients (Figure S9E,F), indicating that this treatment may enhance muscle mass and function. Overall, our findings suggested that the inhibition of inflammation‐mediated RUNX2 activation through prednisolone effectively reduces muscle fibrosis and improves muscle function in DMD patients.

## 3 Discussion

Muscle wasting and weakness are significant consequences of aging and MD, severely impacting overall health and leading to poor outcomes. Chronic inflammation and fibrosis are established pathological hallmarks of MD [7, 46, 47]. In this study, we identified a novel role of *RUNX2* in regulating muscle fibrosis by linking macrophages and the FAP niche in muscles. Specifically, we revealed a new role for the chemokine‐TGF‐β1‐RUNX2 axis in determining FAP differentiation and modulating muscle fibrosis in both humans and mice. We demonstrated that muscle FAP‐derived RUNX2 is activated in response to inflammatory cytokine stimulation. Importantly, specific deletion of *RUNX2* in muscle FAPs resulted in reduced fibrosis in a rodent model of MD. From a therapeutic perspective, pharmacological inhibition of RUNX2 effectively improved dystrophic muscle integrity by attenuating fibrosis. Given that systemic administration of CADD522 may exert effects beyond skeletal muscle, future studies should employ muscle‐targeted delivery strategies to further delineate its tissue‐specific actions. Collectively, these findings demonstrate that RUNX2 mediates the influence of inflammatory cytokines on FAP differentiation and fibrotic progression, thereby establishing a direct molecular link between inflammation and fibrosis in MD.

The immune system plays a crucial role in various physiological processes, including skeletal muscle damage and repair [48]. While inflammation serves as a primary defence mechanism in mammalian cells, the specific inflammatory mediators involved in muscle injury and regeneration are still under investigation. Inflammatory cytokines are essential for muscle repair following injury, as they activate FAPs and muscle satellite cells, which are both key components of regeneration [49, 50, 51]. Conversely, some studies suggest that pro‐inflammatory cytokines may trigger a fibrotic response, leading to FAP activation and increased production of fibronectin and collagen [52, 53]. However, the detailed molecular mechanisms of muscle inflammation‐mediated fibrosis in MD have not yet been fully understood. Our study demonstrated that macrophages release excessive amounts of TGF‐β1, which stimulates RUNX2 activation in FAPs and their differentiation into fibrotic cells that produce ECM components in dystrophic muscles. Furthermore, we demonstrated that a chemokine‐driven inflammatory response contributes to FAP activation and subsequent muscle fibrosis. Thus, we demonstrated a novel mechanism involving the TGF‐β1‐RUNX2 axis that promotes inflammation‐mediated muscle fibrosis in MD.

Building upon its established roles in bone formation and liver fibrosis [40, 54], we identified a novel function for RUNX2 in the skeletal muscle: determining FAP fate within skeletal muscle and regulating muscle fibrosis. A recent study indicated that mice lacking both *RUNX1* and *RUNX2* in CXC chemokine ligand 12‐abundant reticular cells exhibited increased bone marrow fibrosis, suggesting antifibrotic roles of *RUNX1* and *RUNX2* in that context [55]. An increasing body of evidence has shown that *RUNX2* is a critical transcription factor involved in promoting fibrosis across various tissues and cell types [40, 41]. Its role in mediating fibrotic processes in cardiovascular, hepatic, and pulmonary diseases highlights its therapeutic potential as a target for managing fibrosis‐related conditions [40, 41, 56]. While RUNX2 is known to directly regulate both the Fgf signalling pathway (specifically *Fgfr2* and *Fgfr3*) and the Wnt signalling pathway (including *Tcf7*, *Wnt10b*, and *Wnt1*), our data suggested a mechanism by which RUNX2 participates in muscle fibrosis in a way that may be independent of these pathways. We found that RUNX2 directly activates the transcription of both the *Col1a1* and *FN1* genes. Specifically, we identified a unique *RUNX2* binding site in the mouse *Col1a1* gene, conserved in both mice and humans and located 1423 bp downstream of the transcription start site, which is responsible for *RUNX2*‐mediated transcriptional activation of the *Col1a1* gene. Importantly, we found inhibition of RUNX2‐mediated muscle fibrosis, increased myofiber CSA, and improved overall muscle function. Therefore, further elucidating the role of RUNX2 in fibrosis across different cellular contexts and related mechanisms is crucial for developing effective clinical therapies.

Our study explored the crosstalk between myofibers, macrophages, and FAPs, elucidating its role in controlling FAP fate through the chemokine‐TGF‐β1‐RUNX2 axis. This study broadens the scope of research beyond the previously established interactions of macrophages and myofibers with FAPs in the regulation of muscle fibrosis [16, 31, 50, 57]. FAPs are key responders to muscle injury and orchestrate the repair process through dynamic interactions with macrophages and muscle stem cells [23, 54]. We identified a new pathway whereby macrophages modulate FAP function via the release of TGF‐β1, thereby determining RUNX2‐mediated FAP fate and regulating muscle fibrosis. Consistent with previous studies, we found that muscle‐derived CCL2 and CCL7 are major chemokines involved in the recruitment of monocytes/macrophages and TGF‐β1 secretion [30, 39]. Elevated TGF‐β1 in turn promotes fibroblast‐to‐myofibroblast differentiation, thereby linking chemokine‐driven macrophage infiltration to the development of muscle fibrosis. Overall, this study revealed that a novel chemokine‐TGF‐β1‐RUNX2 axis mediates myofiber–macrophage–FAP crosstalk to regulate muscle fibrosis, representing a promising direction for future research.

In summary, our study highlighted the pivotal role of RUNX2 in controlling the fate of FAPs and facilitating the production of fibrotic tissue. We revealed a novel RUNX2‐orchestrated molecular pathway that coordinates communication between myofibers, macrophages, and the FAP niche. Our findings demonstrated how injured myofibers drive FAP differentiation through the recruitment of macrophages and the release of inflammatory cytokines. Given that suppressing muscle inflammation and fibrosis is vital for alleviating atrophy in MD patients, targeting RUNX2 in FAPs may represent a novel and promising therapeutic strategy for managing MD.

## 4 Methods

### 4.1 Animal Studies

Dysf‐KO mice (strain number T028412) and dystrophin‐deficient C57BL/10ScSnJNju‐Dmdem3Cd4/Gpt (*mdx*) mice (strain number T003035) were acquired from GemPharmatech in Nanjing, China. *Pdgfrα*‐CreER^T2^ (#032770) and Rosa26RmT/mG (B6.129(Cg)‐*Gt (ROSA)26Sor^4(ACTB–tdTomato,–EGFP)Luo^*/J (#007676) mice were acquired from The Jackson Laboratory. The mice were randomly divided into groups for the different analyses. They were housed in plastic cages, at a controlled temperature of 22°C ± 2°C on a 12 h light‒dark cycle and given unrestricted access to pellet food and water. The mice were kept in a specific pathogen‐free environment at Anhui Medical University. Tamoxifen was administered at a dosage of 150 mg/kg body weight for five consecutive days, and the chase was initiated at 6 weeks of age.

The 12‐week‐old mdx mouse model exhibits well‐established pathological hallmarks of muscular dystrophy in the diaphragm, as previously reported by our group [14]. Twelve male *mdx* mice aged 12 weeks were randomly allocated to the vehicle or CADD522 group. Six WT C57BL/10ScSn/J mice aged 12 weeks were used as the control group. Twelve male Dysf‐KO mice aged 6‒8 weeks were randomly allocated to the vehicle or CADD522 group. Six WT C57BL/6J mice aged 6‒8 weeks were used as the control group. CADD522 was purchased from MedChemExpress (Shanghai, China). CADD522 (10 mg/kg, twice a week) was intraperitoneally injected into the mice in the CADD522 group for 4‒6 weeks, while mice in the control, *mdx*, and Dysf‐KO groups were injected with vehicle. At the end of the drug treatment period, all the mice were sacrificed, and blood samples and DIA muscle tissues were collected.

### 4.2 Human Studies

Muscle biopsies were collected from the biceps during pathological assessments, after which they were either prepared for histological examination or immediately frozen in liquid nitrogen for subsequent RNA extraction. Healthy subjects and DMD or LGMD patients were recruited from Children's Hospital of Fudan University (Shanghai, China) and the First Affiliated Hospital of Anhui Medical University (Anhui, China) for the analysis of CK and LDH levels in human serum. These DMD patients and those of the control group were aged 4–8. These LGMD patients and those of the control group were aged 8 to 15. Patients with current or previous liver diseases or other muscle diseases, as diagnosed by the Children's Hospital of Fudan University or The First Affiliated Hospital of Anhui Medical University, were excluded from the present study. Detailed information on patient characteristics was included in Table S7. After overnight fasting, blood samples were collected by centrifugation at 3000 rpm for 5 min. The plasma samples were stored at −80°C until use. Serum CK activity and plasma LDH levels were measured via a HITACHI7080 Automatic Clinical Analyzer (Tokyo, Japan) at the Children's Hospital of Fudan University.

### 4.3 Primary Cell Culture

Primary cells were isolated from the GAS muscles of 5‐week‐old male mice as previously described [8, 14]. Briefly, the muscle tissues were chopped into small pieces (1–2 mm) and incubated in a digestion buffer consisting of collagenase type I (0.5 mg/mL) and dispase (0.25 mg/mL) in Hank's balanced salt solution (HBSS) supplemented with calcium and magnesium for 1.5 h at 37°C with shaking. Then, the cell suspensions were filtered through a 70 µm cell strainer to remove undigested tissue debris. Primary FAPs and satellite cells were sorted with the following monoclonal primary antibodies: anti‐CD31 (553373, BD Pharmingen, 1:200), anti‐α7 integrin (produced in‐house, diluted 1:200) and anti‐PDFGR (AF1042, R&D Systems, diluted 1:100). CD31 (‐), α7 integrin (‐) and PDFGR (+) cells were identified as FAPs, whereas CD31 (‐), α7 integrin (+) and PDFGR (‐) cells were identified as satellite cells. Macrophages were sorted with the following monoclonal primary antibodies: anti‐CD45 (147708, BioLegend, 1:500), anti‐CD11b (101230, BioLegend, diluted 1:500), and anti‐F4/80 (111704, BioLegend, diluted 1:500). CD45 (+), CD11b (+), F4/80 (+) cells were identified as macrophages. The sorted FAPs were subsequently plated in culture dishes coated with fibronectin. The FAPs were then cultured in growth medium supplemented with 10% FBS containing basic fibroblast growth factor (bFGF) and insulin‐like growth factor (IGF). The sorted satellite cells were plated in culture dishes coated with 1% Matrigel. The satellite cells were then cultured in growth medium supplemented with 20% FBS containing fibroblast growth factor and epidermal growth factor. All primary cells were grown in a humidified incubator at 37°C in 5% CO_2_, and all growth media were supplemented with 1000 units/mL penicillin and 100 mg/mL streptomycin.

### 4.4 Primary Myofiber Isolation and Culture

Single myofibers were isolated from mice following a previously published protocol [8]. Briefly, extensor digitorum longus muscles were dissected from 8‐week‐old male mice and transferred to a digestion solution consisting of collagenase type I (0.25 mg/mL) in HBSS supplemented with calcium and magnesium. The tissues were subsequently incubated in the digestion solution at 37°C for 45 min with gentle agitation (60 rpm) to dissociate the muscle fibers. After digestion, the digested muscle suspensions were transferred to a fresh dish containing cold DMEM supplemented with 10% FBS to stop the enzymatic reaction. Under a dissecting microscope, the myofibers were gently teased apart to separate them into single fibers. The single myofibers were then transferred to a new dish containing culture medium for further experiments.

### 4.5 Blood Biochemical Analysis

Blood was collected from the inferior vena cava before the mice were euthanized. The blood samples were centrifuged at 3500 rpm for 10 min at 4°C, and then serum CK and plasma LDH levels were measured via a HITACHI7080 Automatic Clinical Analyzer (Tokyo, Japan).

### 4.6 ELISA

Serum IL‐17B, IL‐34, IL‐1β, TNF‐α, and TGF‐β1 levels were measured via ELISA. Before the assay, the latent form of TGF‐β1 in human or mouse serum was activated to the immunoreactive form; 1 N HCl was used for activation, and 1.2 N NaOH/0.5 m HEPES was used for neutralization. Sandwich ELISA for IL‐17A, IL‐34, IL‐1β, TNF‐α, and TGF‐β1 was performed according to the manufacturer's instructions. The human/mouse ELISA kits were purchased from Thermo Fisher Scientific or Abcam.

### 4.7 Histological Analysis

Mouse muscle tissues were quickly frozen in isopentane cooled in liquid nitrogen. Ten‐micron‐thick serial muscle sections were cut with a Leica CM1950 cryostat at −20°C and mounted on positively charged glass slides. The muscle cross‐sections were stained with hematoxylin & eosin (HE) (Sigma‒Aldrich) according to a standard protocol, the sections were viewed, and photomicrographs were captured under a light microscope (BX53, Olympus, Japan). The muscle fiber size was quantified with NIH ImageJ software (version 1.6.0_24). At least 10 fields of different sections from each mouse sample were quantified. For immunofluorescence staining, muscle cross‐sections were incubated with antibodies against fibronectin (550274, BD Biosciences), collagen I (AF1042, R&D Systems), CD68 (123132, BioLegend), and CD206 (141712, BioLegend). Briefly, the sections were washed with ice‐cold PBS and then fixed in ice‐cold 4% PFA for 10 min. These sections were then blocked with 5% normal goat serum (NGS) in PBS for 45 min at room temperature, followed by incubation with primary antibodies at 4°C overnight. The sections were washed three times with PBS for 5 min each and then sequentially incubated with appropriate secondary antibodies for 1 h at room temperature. The muscle sections were then washed three times with PBS for 5 min each. Images of the stained sections were captured under a confocal scanning microscope (Leica, STED) with LAS X (version 3.5.1.18803). Confocal images were acquired from *n* = 3 muscle cross‐sections per sample, then IF quantification of fibrosis area via ImageJ. The percentage represents the fibrosis area relative to the total area.

### 4.8 AAV Injection

AAV9 vectors containing a control construct or shRNA for in vivo expression were generated by Rongsen Gene Technology Co., Ltd. (Jiangsu, China). Briefly, recombinant AAV9 vectors were generated to induce the expression of shRNA or a control construct under the control of the CMV promoter. AAV9 vectors were subsequently generated via the packaging plasmids pAAV‐helper and pAAV9 together with pAAV‐CMV‐sh*Runx2*, pAAV‐CMV‐shCtrl, pAAV‐VMV‐sh*Ccl2*, or pAAV‐CMV‐sh*Ccl7*. The shRNA sequences were as follows: shRunx2: 5’ AGGTTCAACGATCTGAGATTT’; sh*Ccl2*: 5’ TTTAATGTATGTCTGGACCCA’; and sh*Ccl7*: 5’ GTTTCTTGACATAGCAGCATG’. AAV9 was diluted in 0.9% NaCl at 1 × 10^13^ Vp/mL and injected into the muscles (20 µL per GC muscle) of 2‒3‐week‐old *mdx* mice. AAV9‐shCtrl was used as a control.

### 4.9 RNA‐Seq

Transcriptomic analyses were performed via RNA‐seq as described previously [58]. Total RNA was isolated from the biceps muscles of male patients with LGMD or DMD or from those of healthy subjects via RNAiso Plus (Takara Bio). RNA‐seq was performed on the Illumina HiSeq 4000 platform by Beijing Novogene Bioinformatics Technology Co., Ltd. Two to four independent samples per group were analyzed. Paired‐end and 150 nt reads were obtained from the same sequencing lane. The sequencing reads were then aligned to the UCSC mm10 genome assembly via TopHat 2.0.14 with default parameters. Fragments per kilobase of exon per million mapped reads values were calculated via Cufflinks 2.2.1. The criteria used to define differentially expressed genes in patients versus healthy subjects were a fold change >2 (either direction) and a *p* value indicating statistical significance (< 0.05). GO enrichment analysis was used to interpret the data, and the enriched terms were ranked according to the *p* value. Notably, differences in the expression of genes associated with the chemokine and inflammation‐related signaling pathways identified via RNA‐seq analyses were validated via RT‒qPCR. Heatmap analysis of the differentially expressed genes was performed via R software (version 3.3.2).

### 4.10 Luciferase Reporter Assays

The vectors pSG5, pcDNA3.1, and pcDNA3.1‐Runx2 were used in this experiment and have been previously reported [59]. We isolated the promoter of the mouse *Fn1 and Col1a1* gene through PCR amplification of the genomic DNA of C57BL/6J mice and inserted it into the pGL3 Basic luciferase reporter vector at the restriction sites Xho 1 and Hind III. The following primers were used for the promoter of the mouse *Fn1* gene cloning: forward, 5’ ACGCGTGCTAGCAAAAATTGTCTTCATGCAAGTCCGCTGTT; reverse, 5’ CAGTACCGGAATGAGTGAGGTAGGGTGTCTTCCTTGAACG. The following primers were used for the promoter of the mouse *Col1a1* gene cloning: forward, 5’ ACGCGTGCTAGC ACAAAGGGTGAGCAGACAAGTCCGCTGTT; reverse, 5’ CAGTACCGGAAT AGCTCTCCATCAAGATGGTTCCTTGAACG. Verification of all the constructs was achieved through DNA sequencing with Illumina PE150 (Tsingke Biotech Co., Ltd., Beijing).

### 4.11 ChIP‒qPCR

Chromatin immunoprecipitation was performed via a ChIP assay kit (Thermo Fisher Scientific, 26156) according to the instructions provided by the manufacturer [60]. Briefly, cells were incubated with 1% formaldehyde (Thermo Fisher Scientific, 28908) for 10 min at room temperature to cross‐link the proteins to DNA. After cross‐linking, the chromatin was prepared for digestion with the optimal micrococcal nuclease (New England Biolabs, M2047S). Phenol–chloroform extraction was used to purify the DNA.

### 4.12 RNA Analyses

Quantitative RT‒PCR was performed as described previously. Total RNA was extracted from the DIA or GAS muscle via RNAiso Plus (Takara Bio), and the quantity and quality of the RNA were analyzed via a NanoDrop instrument (NanoDrop Technologies, Wilmington, DE). The purified RNA samples were then reverse transcribed via the PrimeScript RT Reagent Kit with gDNA Eraser (Takara Bio). Real‐time quantitative RT‒PCR was performed on the ABI Prism Step‐One system (Thermo Fisher Scientific, 7900HT) with a reagent kit from Takara Bio. The sequences of the primers for the target gene sequences are listed in Appendix Tables S2 and S3. The levels of target mRNAs in arbitrary units were normalized to the level of HRPT1. Relative gene expression was calculated via the 2‐ΔΔCt method.

### 4.13 Western Blot

Western blotting was performed as described in our previous study [58]. The total protein concentration was measured via a bicinchoninic acid (BCA) assay via the Pierce BCA Assay Kit (Thermo Fischer Scientific). The protein signals were detected with the ChemiDoc Imaging System (Tanon, Shanghai, China), and the expression levels of target proteins were quantified with ImageJ Fiji software and normalized to the expression level of α‐tubulin.

### 4.14 Quantification and Statical Analysis

All statistical analyses were performed using GraphPad Prism v8. Data were tested for normality and homogeneity of variance before analysis. For datasets that met these assumptions, parametric tests were applied, including the two‐tailed Student's *t*‐test for comparisons between two groups, or one‐way ANOVA followed by Fisher's LSD post hoc test for multiple‐group comparisons. For datasets that did not satisfy normality or variance assumptions, the nonparametric Mann–Whitney *U* test was used. Correlations were determined using Pearson's correlation test. No statistical methods were used to predetermine sample sizes; sample sizes (n) are reported in each figure legend. All data points were included in statistical analyses. Data are presented as mean ± SEM, and statistical significance was defined as *p* < 0.05.

## Author Contributions

D.X., P.W., Z.L. and B.S. conceptualized the study. K.Z., Z.Z., A.K., L.Z. and H.C. provided methodology. D.X., K.Z., Y.Z., Z.Z., A.K., L.Z., H.C., and Z.X. provided investigation. X.L., P.W., Z.L. and B.S. supervised the study. D.X., Y.Z. and K.Z. wrote the original draft of the manuscript. X.L., P.W., Z.L. and B.S reviewed and edited the manuscript.

## Conflicts of Interest

The authors declare no conflicts of interest.

## Ethics Statement

The human studies were reviewed and approved by the Ethical Committee of Children's Hospital of Fudan University (NCT numbers 2022–353 and 2023–32) and the Ethical Committee of The First Affiliated Hospital of Anhui Medical University (Institutional Review Board number PJ2024‐08‐44). The detailed participant characteristics can be found in Appendix Table S7. Before participation, comprehensive explanations regarding the study's objectives and the biopsy collection process were provided to all participants or their parents or guardians. Following this, all the participants provided written informed consent. All procedures involving animals were carried out following the ethical standards for animal care established by our institution and were approved by the Institutional Animal Care and Use Committee (IACUC) at Anhui Medical University (Approval number: DWLLPF‐2024010407).

## Supporting information




**Supporting File**: advs73492‐sup‐0001‐SuppMat.docx.

## Data Availability

The transcriptome (RNA‐seq) data generated in this study have been deposited in the Genome Sequence Archive of the National Genomics Data Center, China National Center for Bioinformation/Beijing Institute of Genomics, Chinese Academy of Sciences (GSA‐Human: HRA006474 and HRA007353), which is publicly accessible (https://ngdc.cncb.ac.cn/gsa‐human). All the data and materials associated with this study are presented in the paper or the Supplementary Materials. The transcription factors that may bind to the FN1 or Colla1 promoter were predicted via Ingenuity Pathway Analysis [http://www.ingenuity.com/]. The binding sites of RUNX2 within the FN1 and Colla1 promoters were predicted via the JASPAR database [https://jaspar.genereg.net/]. All other data supporting the findings of this study are available from the corresponding authors upon reasonable request. The public muscular dystrophy‐associated scRNA‐seq dataset is accessible from the GEO database under accession number GSE156498 (https://doi.org/10.1073/pnas.2018391117).
