# Supplementary material for: RUNX2 Activation in Fibro/Adipogenic Progenitors Promotes Muscle Fibrosis in Muscular Dystrophy
Source: Adv Sci (Weinh). 2025 Dec 22;13(13):e10850. doi: 10.1002/advs.202510850 (PMC12955923; doi:10.1002/advs.202510850)
Supplement: Supplementary file 1 — Supporting File: advs73492‐sup‐0001‐SuppMat.docx. [file ADVS-13-e10850-s001.docx]

Supplementary Materials for

**RUNX2 Activation in Fibro/Adipogenic Progenitors Promotes Muscle Fibrosis**

**in Muscular Dystrophy**

Pengkai Wu et al.

Corresponding author: Dengqiu Xu, xudengqiu@nju.edu.cn

**The file includes:**

Supplementary materials

Figs. S1 to S9

Tables S1 to S7

Supplementary materials

| **REAGENT or RESOURCE** | **SOURCE** | **IDENTIFIER** |
| --- | --- | --- |
| Antibodies | | |
| Mouse anti-CD68 | Biolegend | Cat# 333819; RRID: AB_2571962 |
| Rabbit anti-Fibronectin | Abcam | Cat# ab32419; RRID: AB_732379 |
| Rabbit anti-Collagen I | Cell Signaling Technology | Cat# 72026S; RRID: AB_2904565 |
| Mouse anti-Tubulin | Bioworld Technology | Cat# BS1699; RRID: AB_1664134 |
| Rabbit anti-RUNX2 | Cell Signaling Technology | Cat# 12556S; RRID: AB_2732805 |
| Mouse anti-NR2E3 | Santa Cruz Biotechnology | Cat# sc-374513; RRID: AB_10990267 |
| Mouse anti-SOX4 | Santa Cruz Biotechnology | Cat# sc-130633; RRID: AB_2066884 |
| Mouse anti-PAX7 | DSHB | Cat# PAX7; RRID: AB_528428 |
| Mouse anti-eMyHC | DSHB | Cat# F1.652; RRID: AB_528358 |
| Mouse anti-MHC | DSHB | Cat# MF20; RRID: AB_2147781 |
| Rabbit anti-F4/80 | Cell Signaling Technology | Cat# 30325S; RRID: AB_2798990 |
| Rabbit anti-PDGFR-α | Cell Signaling Technology | Cat# 3174T; RRID: AB_2162345 |
| Rabbit anti-CD206 | Cell Signaling Technology | Cat# 24595T; RRID: AB_2892682 |
| Rat anti-CD31 | BD Biosciences | Cat# 553373; RRID: AB_394819 |
| Mouse anti-PDFGR | R&D Systems | Cat# AF1042; RRID: AB_2162633 |
| Alexa Fluor 488 Donkey anti-Rabbit | Yeasen Biotech | Cat# 34206ES60; RRID: AB_2909605 |
| Alexa Fluor 594 Donkey anti-Rabbit | Yeasen Biotech | Cat# 34212ES60; RRID: AB_2920875 |
| Alexa Fluor 594 Goat anti-mouse | Yeasen Biotech | Cat# 33212ES60; RRID: AB_3096072 |
| Goat anti-Mouse IgG (H+L) Secondary Antibody, HRP | Thermo Fisher Scientific | Cat# [31460](https://www.thermofisher.cn/order/catalog/product/cn/en/31460); RRID: AB_228341 |
| Bacterial and virus strains | | |
| AAV-2/9-shCtrls | Rongsen Gene Technology Co., Ltd. | http://www.rongsenbio.com/ |
| AAV-2/9-sh*Runx2* | Rongsen Gene Technology Co., Ltd. | http://www.rongsenbio.com/ |
| AAV-2/9-sh*Ccl2* | Rongsen Gene Technology Co., Ltd. | http://www.rongsenbio.com/ |
| AAV-2/9-sh*Ccl7* | Rongsen Gene Technology Co., Ltd. | http://www.rongsenbio.com/ |
| Biological samples | | |
| Human muscle tissues | Children's Hospital of Fudan University | N/A |
| Chemicals, peptides, and recombinant proteins | | |
| RIPA Lysis Buffer | Beyotime Biotechnology | Cat# P0013B |
| collagenase type I | MedChemExpress | Cat# HY-E70005A |
| dispase | Biosharp | Cat# BL1360A |
| CADD522 | MedChemExpress | Cat# HY-107999 |
| Basic FGF | Sino Biological | Cat# 10014-HNAE |
| IGF1 | MedChemExpress | Cat# HY-P1777 |
| FBS | WISENT | Cat# 086-150 |
| EGF | Sino Biological | Cat# 10605-HNAE |
| HBSS | Beyotime Biotechnology | Cat# C0219 |
| Glycerol | Biosharp | Cat# BS154 |
| NaOH | Aladdin | Cat# S111502 |
| HEPES | Sigma-Aldrich | Cat# H7006 |
| PBS | WISENT | Cat# 311-010-CL |
| DMEM | WISENT | Cat# 319-005CL |
| formaldehyde | Thermo Fisher Scientific | Cat# 28908 |
| micrococcal nuclease | New England Biolabs | Cat# M2047S |
| TGF-β1 | Sino Biological | Cat# 10804-HNAC |
| TNF-α | Sino Biological | Cat# 10602-HNAE |
| IL-1β | Sino Biological | Cat# 10139-HNAE |
| IL-17A | Sino Biological | Cat# 12047-HNAE |
| Critical commercial assays | | |
| BCA protein assay kit | Vazyme Biotechnology | Cat# E112-02 |
| human TGF-β1 kits | Thermo Fisher Scientific | Cat# BMS2065 |
| human IL-34 ELISA kits | Thermo Fisher Scientific | Cat# EH275RB |
| human TNF-α ELISA kits | Thermo Fisher Scientific | Cat# 88-7346-88 |
| human IL-17 ELISA kits | Thermo Fisher Scientific | Cat#88-7176-88 |
| human IL-1β ELISA kits | Thermo Fisher Scientific | Cat# 88-7261-88 |
| mouse IL-17 ELISA kits | Thermo Fisher Scientific | Cat# BMS6001 |
| mouse IL-34 ELISA kits | Abcam | Cat# ab213873 |
| mouse TNF-α ELISA kits | Thermo Fisher Scientific | Cat# BMS607-3 |
| mouse TGF-β1 ELISA kits | Thermo Fisher Scientific | Cat# BMS6001 |
| mouse IL-1β ELISA kits | Thermo Fisher Scientific | Cat# 88-7013-22 |
| Dual-Glo Luciferase Assay | Promega | Cat# N1110 |
| ChIP assay kit | Thermo Fisher Scientific | Cat# 26156 |
| Deposited data | | |
| Human musle RNA seq between LGMD patients and controls | This study | GSA-Human: HRA006474 |
| Human musle RNA seq between DMD patients and controls | This study | GSA-Human: HRA007353 |
| Mouse muscular dystrophy-associated scRNA seq | Wang et al. | GEO: GSE156498 |
| Experimental models: Cell lines | | |
| Mouse: Dysf-KO mice | GemPharmatech in Nanjing, China | strain# T028412 |
| Mouse: mdx mice | GemPharmatech in Nanjing, China | strain# T003035 |
| Mouse: Runx2 flox/flox mice | GemPharmatech in Nanjing, China | strain# T010084 |
| Mouse: PDGFR-α-Cre/ERT2 mice | The Jackson Laboratory | JAX: 032770 |
| Oligonucleotides | | |
| shRNA targeting sequence: Runx2:  AGGTTCAACGATCTGAGATTT | This paper | N/A |
| shRNA targeting sequence: Ccl2:  TTTAATGTATGTCTGGACCCA | This paper | N/A |
| shRNA targeting sequence: Ccl7:  GTTTCTTGACATAGCAGCATG | This paper | N/A |
| Software and algorithms | | |
| ImageJ | Schneider et al., 2012 | https://imagej.nih.gov/ij/ |
| GSEA | Mootha et al, 2003 | https://www.gseamsigdb.org/gsea/index.jsp |
| iProx | Ma et al, 2019 | https://www.iprox.cn |
| Graphpad Prism 8.0 | Swift et al, 1997 | https://www.graphpad.com/ |
| Gene Ontology | Ashburner et al, 2000 | http://geneontology.org/ |


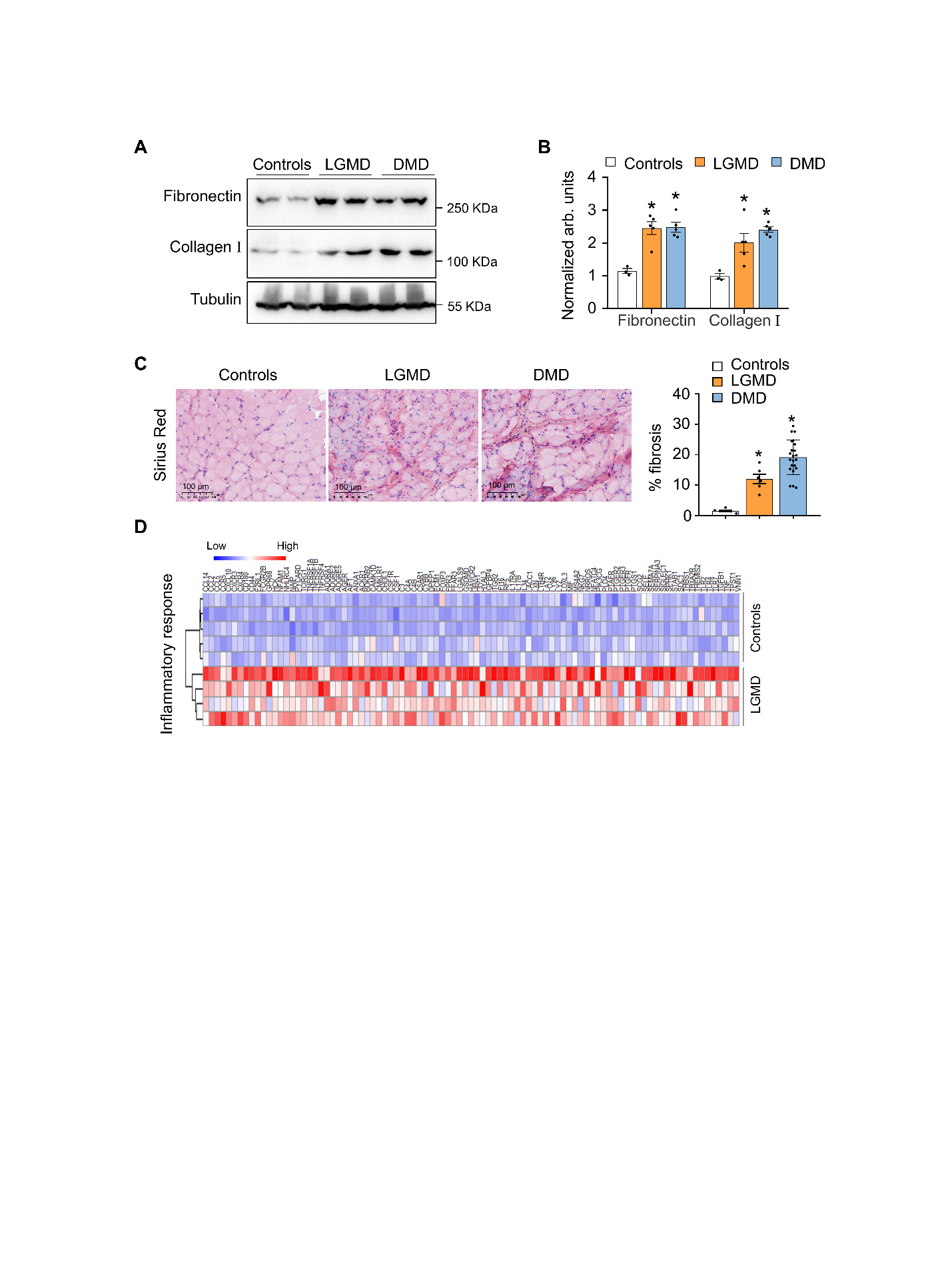
**Figure S1. Pathologic features of muscular dystrophies.**

(A) Western blot analysis showing the levels of Fibronectin and Collagen Ⅰ in human biceps muscles from age-matched male LGMDs patients, DMD patients and normal controls. Normal male, n = 3; Male LGMDs, n = 5; Male DMDs, n = 5.

(B) Quantitative analysis of Fibronectin/Τubulin and Collagen Ⅰ/Τubulin ratios as shown in (A).

(C) Representative images from Picrosirius red staining in skeletal muscle sections from the indicated groups. Scale bar represents 50 μm. n = 6‒22 per group.

(D) Heatmaps presenting the analysis of RNA-seq data for inflammatory response pathway related-genes found to be upregulated in biceps muscles of LGMDs patients.

Values represent mean ± SEM; **P* < 0.05 vs. corresponding controls. *P* values were determined by one-way ANOVA followed by a Fisher’s LSD post-hoc test (B and C).


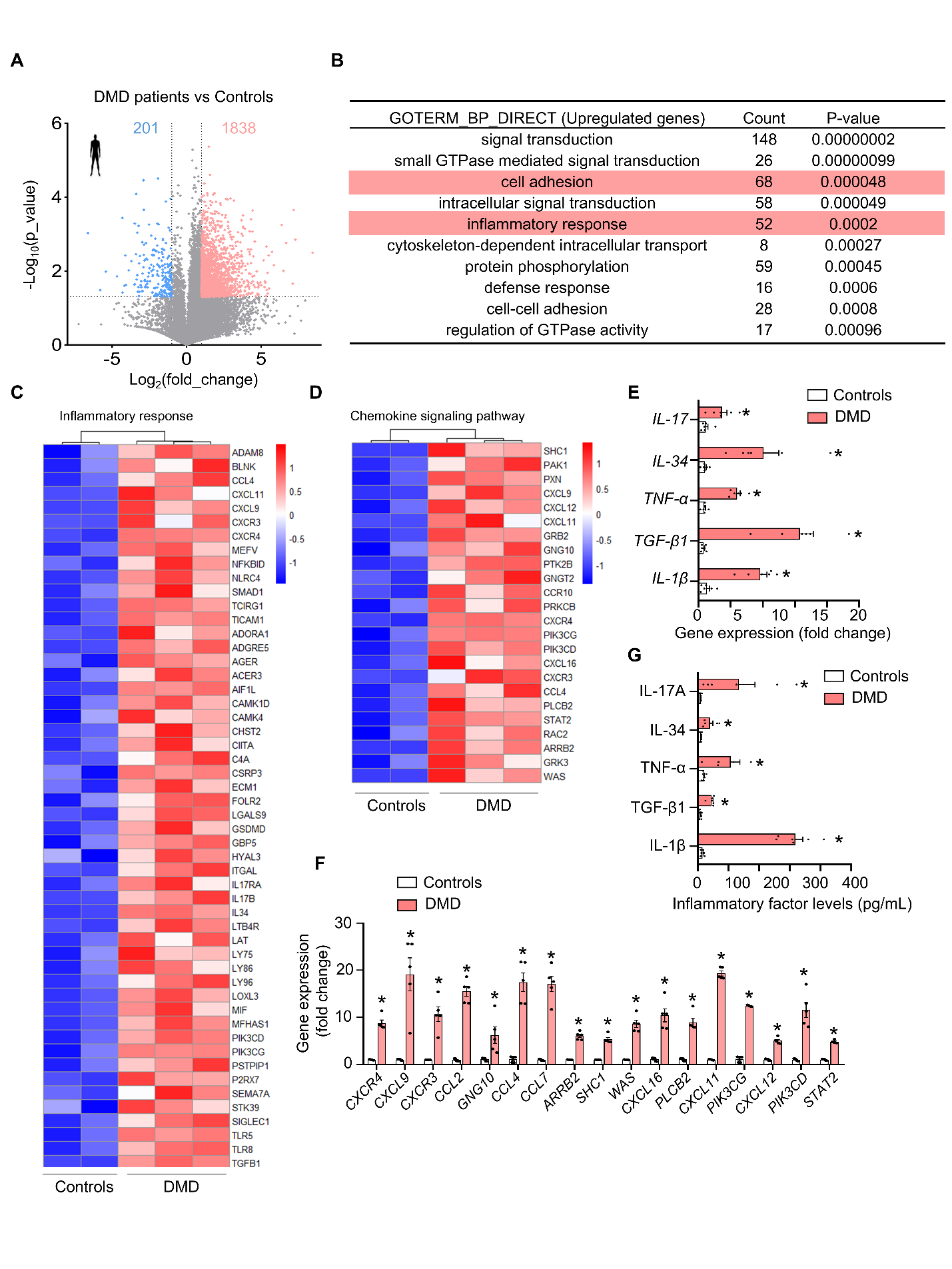


**Figure S2. Chemokine for immune activation and inflammatory factors secretion in DMD patients.**

(A) A volcano plot illustrating the fold changes against *P*-values from RNA-seq data of biceps muscles in aged matched DMD patients compared to controls. Genes significantly upregulated are marked with red dots, while those downregulated are indicated by blue dots. Male DMDs, n = 3; Normal male, n = 2.

(B) Results of the Biological Process (BP) enrichment analysis highlighting 1838 genes upregulated in the biceps muscles of DMDs patients.

(C and D) Heatmaps presenting the analysis of RNA-seq data for genes found to be upregulated in biceps muscles of DMDs patients.

(E) Comparative gene expression analysis (RT-qPCR) of the chemokine signaling pathway between DMDs patients and control subjects in triceps muscles. Male DMDs, n = 5; Normal male, n = 5.

(F) RT-qPCR examination of genes associated with the inflammatory response pathway in biceps muscles of DMDs patients. Male DMDs, n = 5; Normal male, n = 5.

(G) Levels of serum inflammatory markers in DMDs patients versus normal controls. Male DMDs, n = 5; Normal male, n = 5.

Values represent mean ± SEM; **P* < 0.05 vs. corresponding controls. *P* values were determined by one-way ANOVA followed by two-tailed unpaired Student’s t-test.


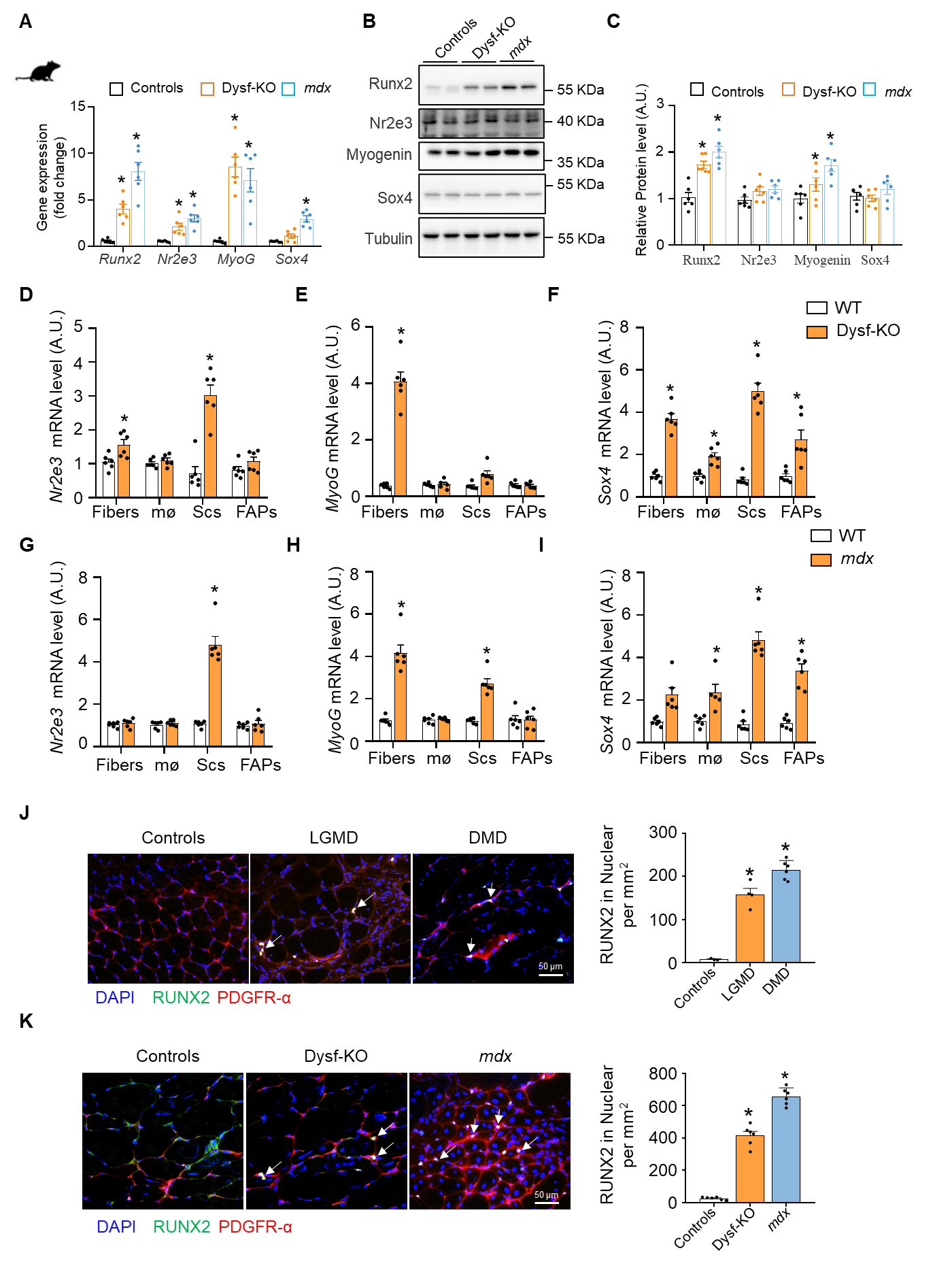


**Figure S3. The mRNA levels of representative transcript factors in single myofibers, satellite cells and FAPs of Dys-KO and mdx mice.**

(A) RT-qPCR examination of the mRNA levels of *Runx2*, *Nr2e3*, *MyoG*, and *Sox4* in the gastrocnemius (GC) muscles of mice models of LGMD, DMD, and WT controls. n = 6 mice per group.

(B and C) Western blot analysis showing the protein levels of Runx2, Nr2e3, Myogenin, and Sox4 in the GC muscles of LGMD, DMD, and WT control mice. n = 6 mice per group.

(D) RT-qPCR analysis of *Nr2e3* in single fibers, satellite cells and FAPs from the skeletal muscle of mdx mice and (G) Dysf-KO mice. n = 6 mice per group.

(E) RT-qPCR analysis of *MyoG* in single fibers, macrophages, satellite cells and FAPs from the skeletal muscle of mdx mice and (H) Dysf-KO mice. n = 6 mice per group.

(F) RT-qPCR analysis of *Sox4* in single fibers, macrophages, satellite cells and FAPs from the skeletal muscle of mdx and (I) Dysf-KO mice. n = 6 mice per group.

(J) Confocal images showing immunostaining for RUNX2 (green) and PDGFR-α (red) in skeletal muscle sections from control individuals, as well as patients with LGMD and DMD. Scale bar represents 50 μm. Male LGMDs, n = 4; DMDs = 6; Normal male, n = 3.

(K) Confocal images showing immunostaining for RUNX2 (green) and PDGFR-α (red) in skeletal muscle sections from controls, Dysf-KO, and mdx mice. Scale bar is 50 μm. n = 6 mice per group.

Data are shown as the mean ± SEM. **P* < 0.05 vs. corresponding controls. *P* values were determined by one-way ANOVA followed by a Fisher’s LSD post-hoc test (C, J and K) and unpaired two-tailed Student’s t-test (D‒I).


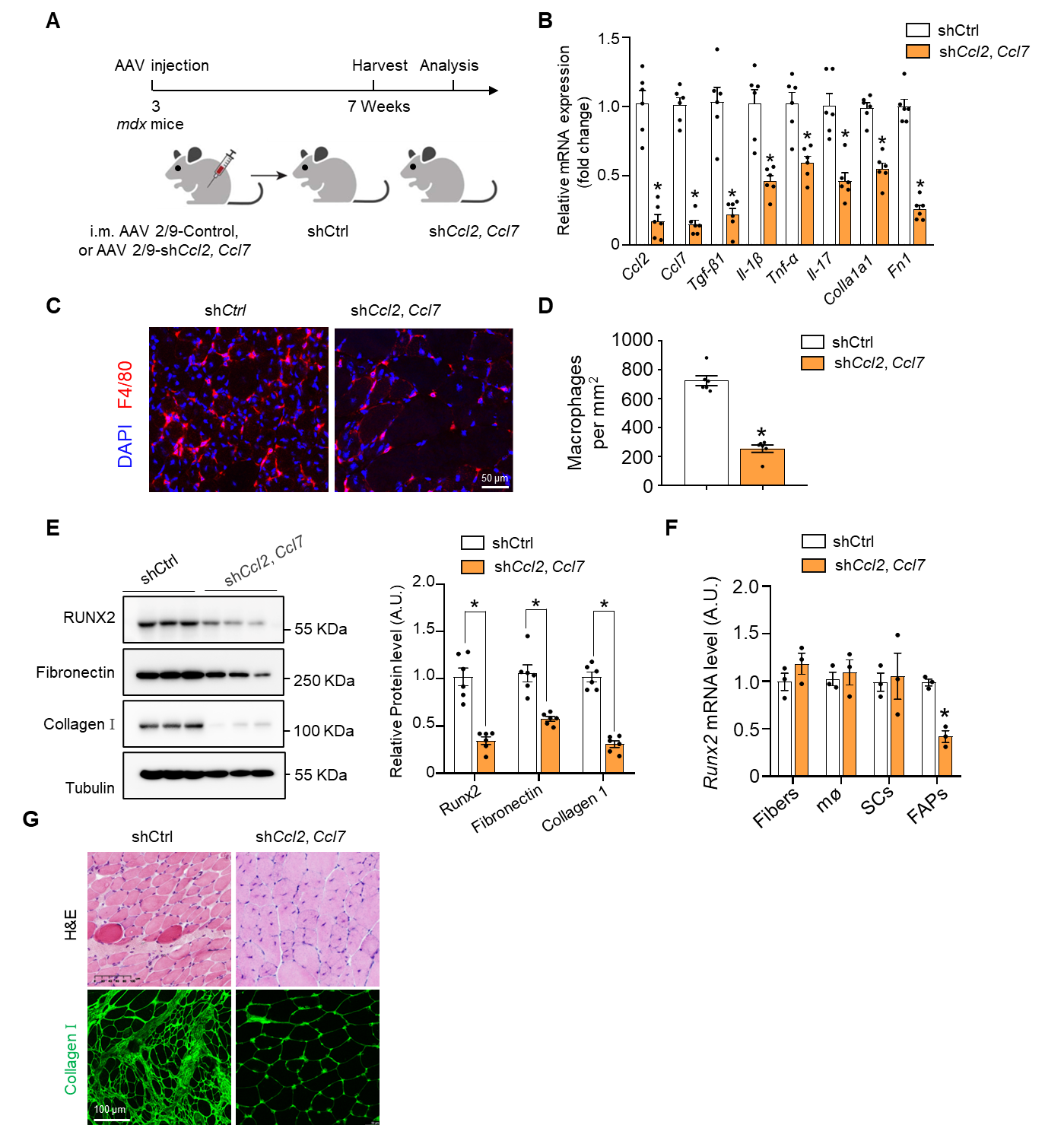


**Figure S4. Ablation of *Ccl2* and *Ccl7* alleviated muscle fibrosis in *mdx* mice.**

(A) AAV9-mediated *Ccl2* and *Ccl7* knockdown in the GAS muscles of *mdx* mice using AAV9-mediated delivery. Briefly, 3-week-old *mdx* mice were given a single intramuscular injection of AAV 2/9-control or AAV-2/9-sh*Ccl2*, *Ccl7* (40 μL, 1*10^11 vg per mouse). Tissue samples were collected 4 weeks post-injection for analysis. n = 6 mice per group.

(B) RT‒qPCR analysis of *Ccl2*, *Ccl7*, *TGF-β1*, *IL-1β*, *TNF-α*, *IL-17*, Colla1a1, and *Fn1* expression in the GAS muscles of *mdx* mice. n = 6 mice per group.

(C) Representative images of F4/80 staining in the GAS muscles of *mdx* mice. The scale bar represents 50 μm. n = 6 per group.

(D) Quantification of the number of F4/80-positive macrophages per mm^2^. n = 6 per group.

(E: left) Representative immunoblot analysis of total protein from the GAS muscles of the indicated *mdx* mice. (E: right) Quantification of the RUNX2/Tubulin, fibronectin/Tubulin, and collagen Ⅰ/Tubulin signal ratios in each group normalized to that in the control group. n = 6 mice per group.

(F) RT-qPCR analysis of *ANXA2* in single fibers, macrophages, satellite cells and FAPs from the skeletal muscle of indicated mice. n = 3 mice per group.

(G) Representative HE-stained images and collagen Ⅰ-stained images of GAS muscles from the indicated *mdx* mice. TSA amplification was used and can exaggerate the collagen signal. The scale bar represents 100 μm. n = 6 mice per group.

The data are shown as the means ± SEMs. **P* < 0.05 vs. the corresponding controls. *P* values were determined by one-way ANOVA followed by a Fisher’s LSD post-hoc test (D) and unpaired two-tailed Student’s t test (B, E and F).


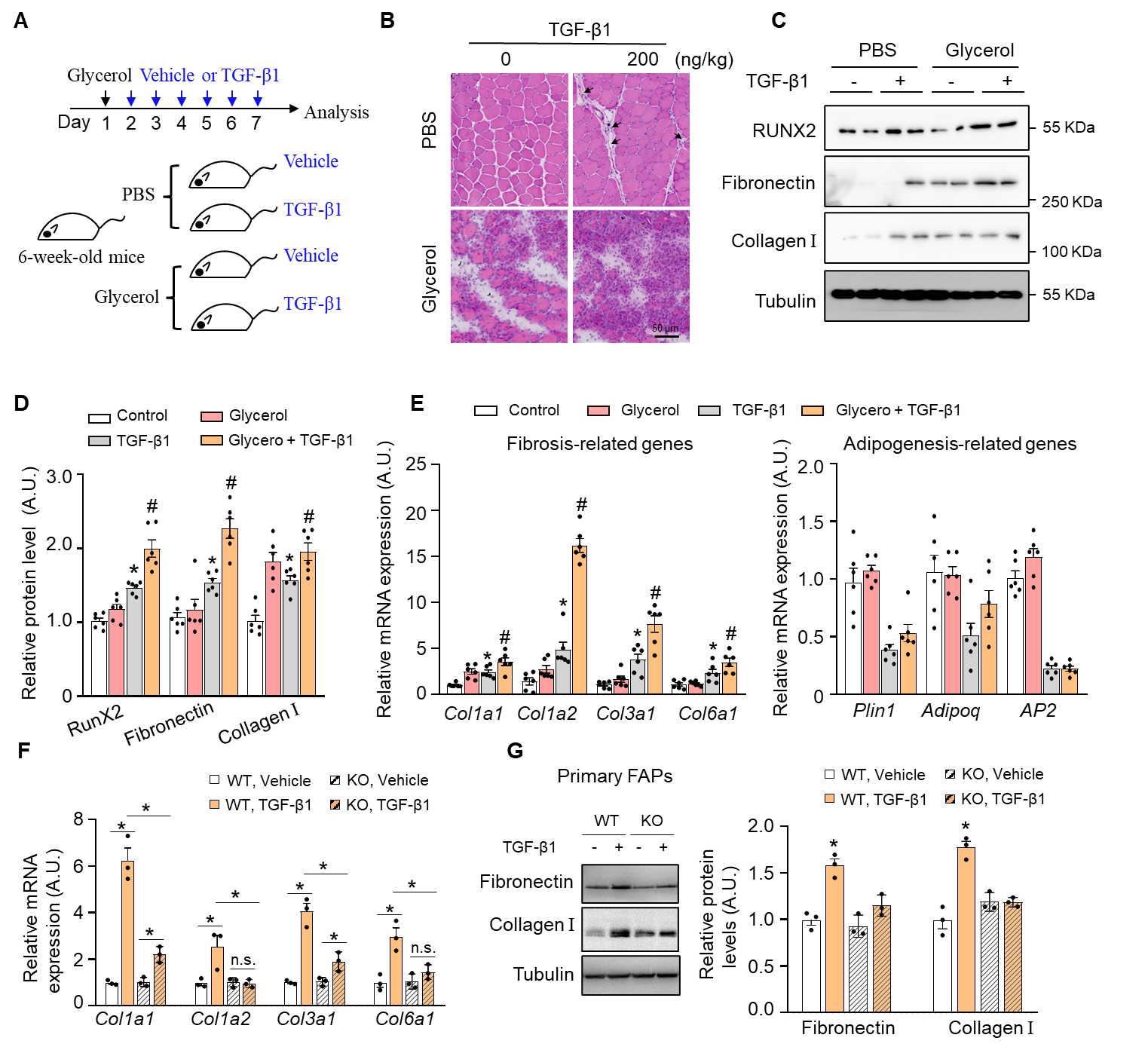


**Figure S5. TGF-β1 promotes RUNX2 activation and fibrotic genes expression in *mdx* mice.**

(A) A schematic diagram depicting the glycerol-induced muscle injury model. Briefly, 6-week-old mice were given a single intramuscular injection of 40 μL of 50% glycerol. Following this, they received daily intraperitoneal injections of TGF-β1 (200 ng/kg) for 7 consecutive days.

(B) Representative images of H&E staining of TA from control mice and glycerol treat-mice mice with or without TGF-β1 treatment.

(C) Western blot analysis of RUNX2, Fibronectin and Collagen Ⅰ protein levels from the indicated mice.

(D) Quantification of the Runx2/Tubulin, Fibronectin/Tubulin and Collagen Ⅰ/Tubulin were normalized to controls. n = 6 mice per group.

(E) RT-qPCR analysis of fibrotic genes, adipogenic genes and muscle-specific genes from the indicated mice. n = 6 mice per group.

(F) RT-qPCR analysis showing the mRNA levels of *Col1a1*, *Col1a2*, *Col3a1* and *Col6a1* in the indicted group.

(G) Western blot analysis showing the protein levels of Fibronectin and collagen Ⅰ in the indicted group.

Data are shown as the mean ± SEM. **P* < 0.05 vs. corresponding controls. # *P* < 0.05 vs. TGF-β group. *P* values were determined by one-way ANOVA followed by a Fisher’s LSD post-hoc test (D, E, F and G).


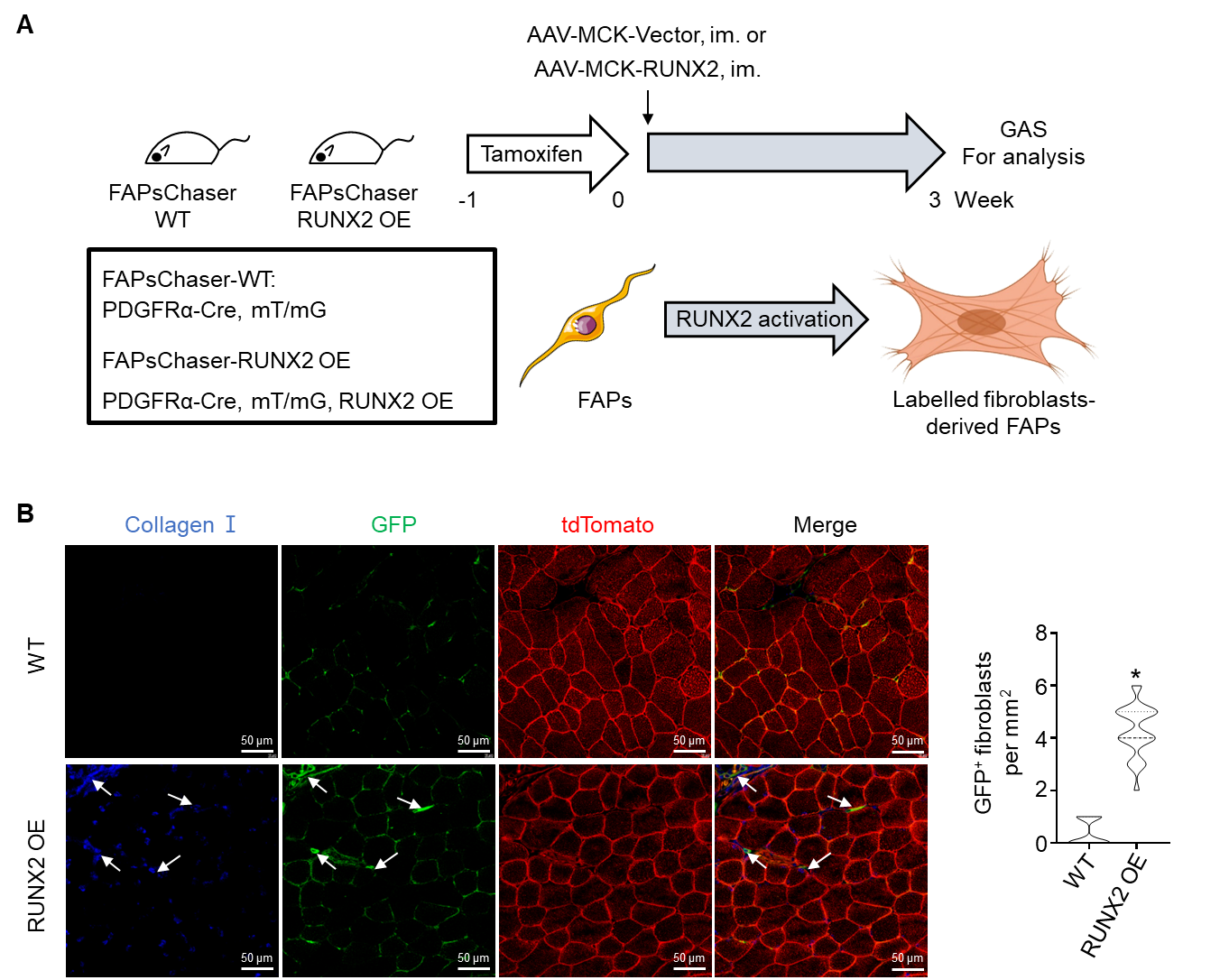


**Figure S6. Overexpression of RUNX2 promotes FAPs differentiate into collagen-producing fibroblasts.**

(A) The FAPsChaser system consists of the PDGFRα-Cre^ERT^ transgene and the CRE-dependent Rosa26RmT/mG reporter allele, which enables permanent labelling of FAPs-expressing fibroblasts with membrane-bound GFP in a Tamoxifen-dependent manner. The mice were treated with Tamoxifen for 7 days. Then, AAV9-mediated RUNX2 overexpression in the GAS muscles of FAPsChaser mice.

(B) GAS sections stained with anti-GFP (green) and anti-Collagen (blue) antibodies. Images are representative of more than 24 fields (n = 3 mice per group). The scale bar represents 50 μm.


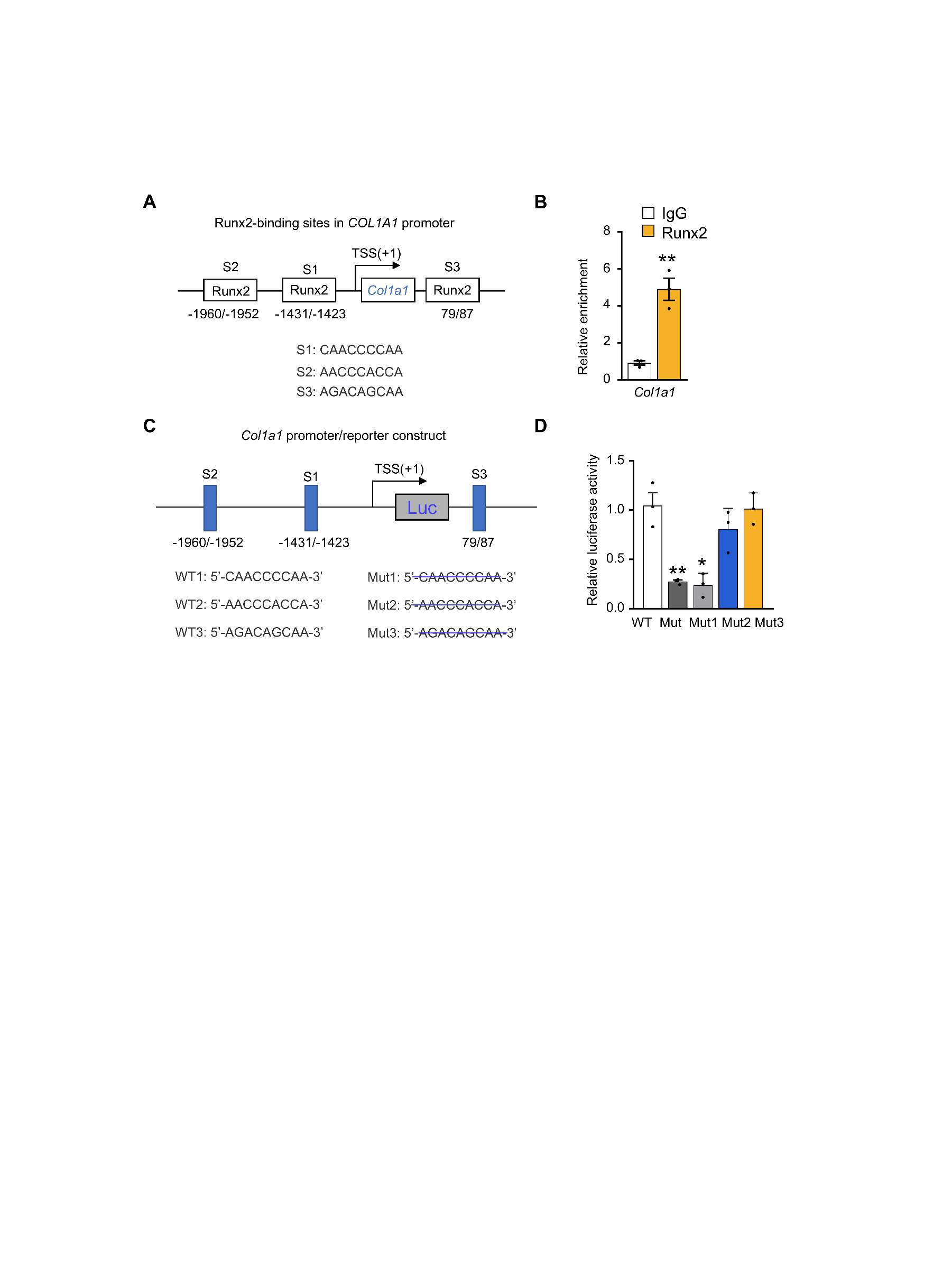


**Figure S7. RUNX2 mediated *Col1a1* gene expression.**

(A) The predicted binding sites of RUNX2 in *Col1a1* promoter according to JASPAR database.

(B) ChIP-qPCR assay in primary FAPs for Runx2 or IgG occupancy at *Col1a1* promoter fragments. n = 3 independent experiments.

(C) Generation of luciferase reporters governed by *Col1a1* promoter with wildtype or mutant Runx2 binding sites.

(D) Dual luciferase reporter assay in primary FAPs co-transduced with luciferase reporter driven by wild-type or mutated promoter, and expression plasmid for Runx2. n = 3 independent experiments.

Data are shown as the mean ± SEM. **P* < 0.05, ***P* < 0.01 vs. corresponding controls or Vector controls. *P* values were determined by one-way ANOVA followed by a Fisher’s LSD post-hoc test (D) and unpaired two-tailed Student’s t-test (B).


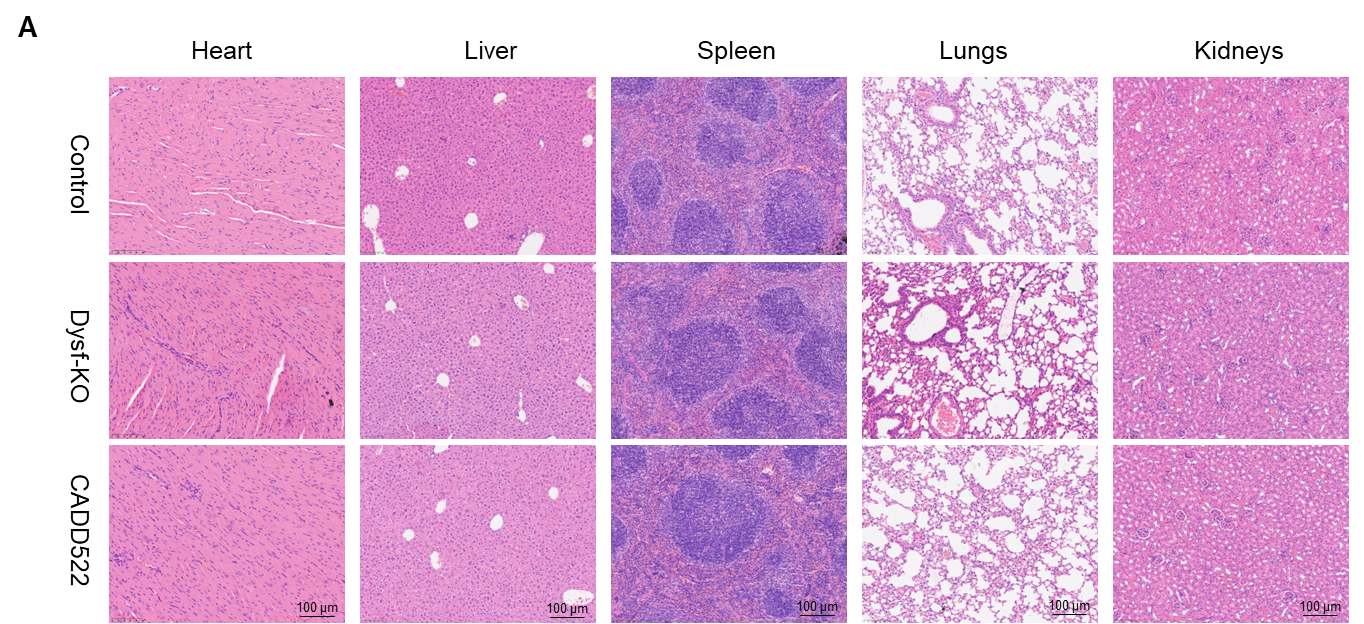


**Figure S8. The toxicity effect of CADD522 on mice.**

(A) 6‒8 weeks male Dysf-KO mice were treated with vehicle or CADD522 (10 mg/kg) for 4 weeks. Representative images of H&E-stained heart, liver, spleen, lungs, and kidneys from the indicated mice. The scale bar represents 100 μm. n = 6 mice per group.


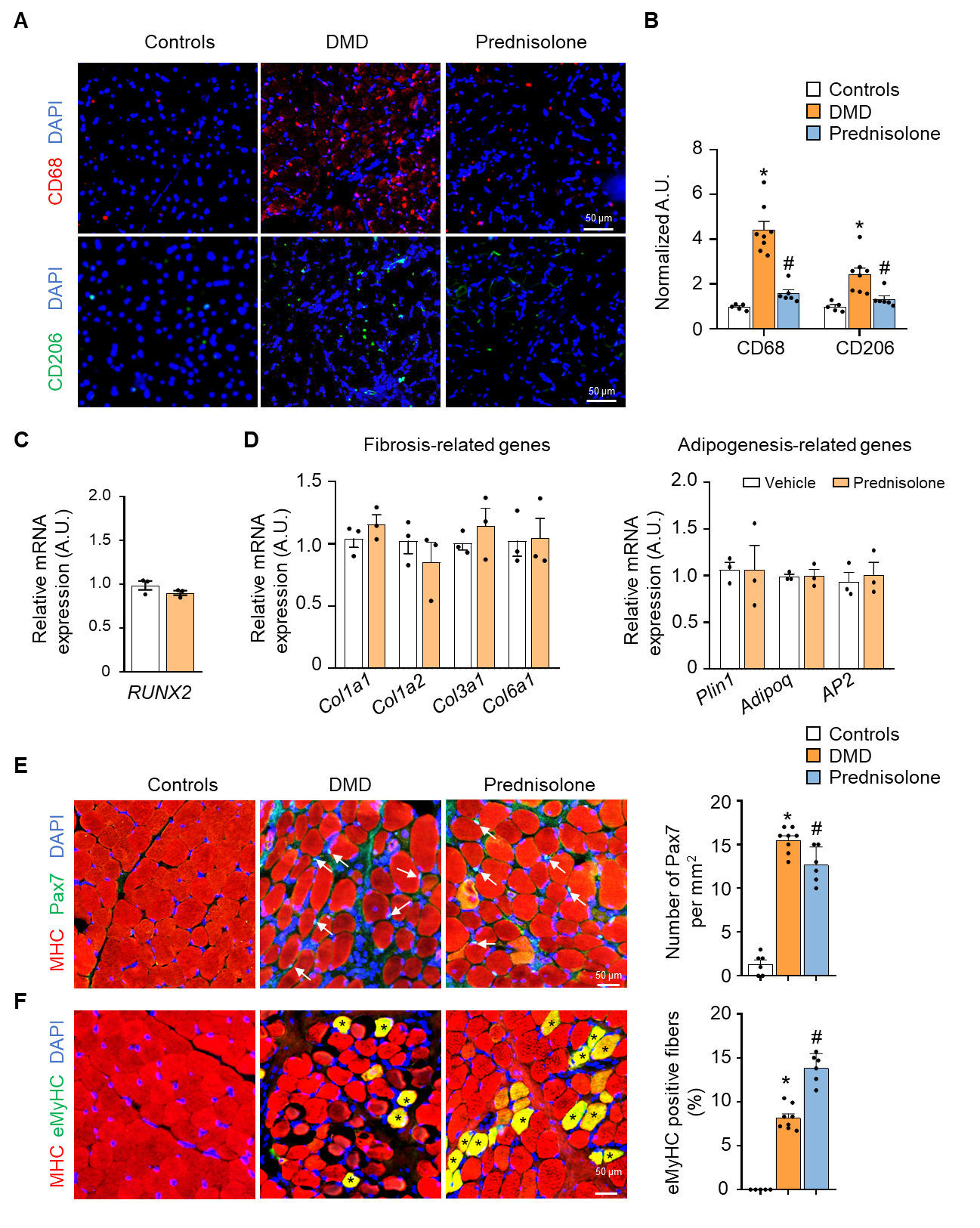


**Figure S9. Prednisolone reduces muscle inflammations.**

(A) Representative immunofluorescent image of CD206 (green) and DAPI (blue). Scale bar represents 50 μm. n = 5‒8 per group.

(B) Quantification of CD68 or CD206 positive cells in muscle cross sections of indicated human subjects. n = 5‒8 per group.

(C)RT‒qPCR analysis of the expression of *Runx2* in isolated FAPs after treatment with 1μM prednisolone.

(D) RT‒qPCR analysis of the expression of fibrosis-related genes and adipogenesis-related genes in isolated FAPs after treatment with 1μM prednisolone.

(E) Representative immunofluorescent image of Pax7 (green), MHC (red) and DAPI (blue), and quantification of satellites in muscle cross sections of indicated human subjects. Scale bar represents 50 μm. n = 5‒8 per group. White arrows indicate stellate cells.

(F) Representative immunofluorescent image of eMyHC (green), MHC (red) and DAPI (blue), and quantification the percentage of eMyHC positive fibers. Scale bar represents 50 μm. n = 5‒8 per group. Asterisks indicate embryonic myosin heavy chain.

Data are shown as the mean ± SEM. **P* < 0.05 vs. corresponding controls. *P* values were determined by one-way ANOVA followed by a Fisher’s LSD post-hoc test.

**Table S1. Genotyping**

| **Table S1 RT-PCR primers** | | |
| --- | --- | --- |
| ***Genotyping*** | **Forward** | **Reverse** |
| *Runx2^f/f^* | 5’- TGCTTGGCGGTGGCGGACA | 5’- CGACTGGAGACCGGACAGAGTC |
| Pdgfrα-CreERT2 | 5’-ATCGCATTCCTTGCA AAAGT | 5’- AGGCCCACAGAACATGGA C |

**Table S2. RT-PCR primers.**

| **Table S2 RT-PCR primers** | | |
| --- | --- | --- |
| ***Mouse Gene*** | **Forward** | **Reverse** |
| *36b4* | 5’-ATCCCTGACGCACCGCCGTGA | 5’-TGCATCTGCTTGGAGCCCACGT |
| *Il-17A* | *5’-TTTAACTCCCTTGGCGCAAAA* | *5’-CTTTCCCTCCGCATTGACAC* |
| *Il-34* | *5’-TTGCTGTAAACAAAGCCCCAT* | *5’-CCGAGACAAAGGGTACACATTT* |
| *Tnf-α* | *5’-CCCTCACACTCAGATCATCTTCT* | *5’-GCTACGACGTGGGCTACAG* |
| *Tgf-β* | *5’-ACCAAGGAGACGGAATAC* | *5’-TGTGGAGCTGAAGCAATA* |
| *Il-1β* | *5’-GCAACTGTTCCTGAACTCAACT* | *5’-ATCTTTTGGGGTCCGTCAACT* |
| *Runx2* | *5’-ATGCTTCATTCGCCTCACAAA* | *5’-GCACTCACTGACTCGGTTGG* |
| *Nr2e3* | *5’-CAGTGGGAAACATTATGGCATCT* | *5’-ACTGATTGCGATGGGCCTTAT* |
| *MyoG* | *5’-GAGACATCCCCCTATTTCTACCA* | *5’-GCTCAGTCCGCTCATAGCC* |
| *Sox4* | *5’-CGGCTGCATCGTTCTCTCC* | *5’-GGTAGACGTGCTTCACTTTCTTG* |
| *Colla1a1* | *5’-GCTCCTCTTAGGGGCCACT* | *5’-CCACGTCTCACCATTGGGG* |
| *Fn1* | *5’-ATGTGGACCCCTCCTGATAGT* | *5’-GCCCAGTGATTTCAGCAAAGG* |
| *Cxcl9* | *5’-TCCTTTTGGGCATCATCTTCC* | *5’-TTTGTAGTGGATCGTGCCTCG* |
| *Cxcl11* | *5’-GGCTTCCTTATGTTCAAACAGGG* | *5’-GCCGTTACTCGGGTAAATTACA* |
| *Ppargc1a* | *5’-TATGGAGTGACATAGAGTGTGCT* | *5’-CCACTTCAATCCACCCAGAAAG* |
| *Col1a2* | *5’-GTAACTTCGTGCCTAGCAACA* | *5’-CCTTTGTCAGAATACTGAGCAGC* |
| *Col3a1* | *5’-ACGTAGATGAATTGGGATGCAG* | *5’-GGGTTGGGGCAGTCTAGTG* |
| *Plin1* | *5’-GGGACCTGTGAGTGCTTCC* | *5’-GTATTGAAGAGCCGGGATCTTTT* |
| *Adipoq* | *5’-TGTTCCTCTTAATCCTGCCCA* | *5’-CCAACCTGCACAAGTTCCCTT* |
| *AP2* | *5’-AAGGTGAAGAGCATCATAACCCT* | *5’-TCACGCCTTTCATAACACATTCC* |
| *MyoD* | *5’-CCACTCCGGGACATAGACTTG* | *5’-AAAAGCGCAGGTCTGGTGAG* |
| *Myf5* | *5’-AAGGCTCCTGTATCCCCTCAC* | *5’-TGACCTTCTTCAGGCGTCTAC* |
| ***Human Gene*** | **Forward** | **Reverse** |
| *HRPT1* | 5’-TATGGCGACCCGCAGCCCT | 5’-CATCTCGAGCAAGACGTTCAG |
| *IL-17A* | 5’-TCCCACGAAATCCAGGATGC | 5’-GGATGTTCAGGTTGACCATCAC |
| *IL-34* | 5’-AAGGTGGAATCCGTGTTGTCC | 5’-AGCTTTGTTTACAGCAGGAGC |
| *TNF-α* | 5’-CCTCTCTCTAATCAGCCCTCTG | 5’-GAGGACCTGGGAGTAGATGAG |
| *TGF-β* | 5’-GGCCAGATCCTGTCCAAGC | 5’-GTGGGTTTCCACCATTAGCAC |
| *IL-1β* | 5’-AGCTACGAATCTCCGACCAC | 5’-CGTTATCCCATGTGTCGAAGAA |
| *RUNX2* | 5’-TGGTTACTGTCATGGCGGGTA | 5’-TCTCAGATCGTTGAACCTTGCTA |
| *NR2E3* | 5’-AGCAGCGGGAAGCACTATG | 5’-CCTGGCACCTGTAGATGAGC |
| *MYOG* | 5’-GGGGAAAACTACCTGCCTGTC | 5’-AGGCGCTCGATGTACTGGAT |
| *SOX4* | 5’-AGCGACAAGATCCCTTTCATTC | 5’-CGTTGCCGGACTTCACCTT |
| *CXCR4* | 5’-ACTACACCGAGGAAATGGGCT | 5’-CCCACAATGCCAGTTAAGAAGA |
| *CXCL9* | 5’-CCAGTAGTGAGAAAGGGTCGC | 5’-AGGGCTTGGGGCAAATTGTT |
| *CXCR3* | 5’-CCACCTAGCTGTAGCAGACAC | 5’-AGGGCTCCTGCGTAGAAGTT |
| *GNGT2* | 5’-AGCGGGAAAGGAAATCAAGGA | 5’-CAGACAGCCACCTTTCTCCTTG |
| *GNG10* | 5’-CCTGCCCTTCACATCGAAGAT | 5’-CTTCTTTGCGAAGCTGCTCAA |
| *CCL4* | 5’-CTGTGCTGATCCCAGTGAATC | 5’-TCAGTTCAGTTCCAGGTCATACA |
| *CCR10* | 5’-TGAAGAGGACGCATACTCGG | 5’-CCACGGTCAGGGAGACACT |
| *ARRB2* | 5’-TCCATGCTCCGTCACACTG | 5’-ACAGAAGGCTCGAATCTCAAAG |
| *SHC1* | 5’-GCCAAAGACCCTGTGAATCAG | 5’-GTATTGTTTGAAGCGCAACTCG |
| *WAS* | 5’-GATGCTTGGACGAAAATGCTTG | 5’-CCCCACAATGCTCCTTGGT |
| *CXCL16* | 5’-GACATGCTTACTCGGGGATTG | 5’-GGACAGTGATCCTACTGGGAG |
| *PLCB2* | 5’-GTCTGGTCTGAGGAGCTATTCA | 5’-GCTTCAGCTTCGTGTATGCTT |
| *CXCL11* | 5’-GACGCTGTCTTTGCATAGGC | 5’-GGATTTAGGCATCGTTGTCCTTT |
| *PIK3CG* | 5’-GGCGAAACGCCCATCAAAAA | 5’-GACTCCCGTGCAGTCATCC |
| *CXCL12* | 5’-ATTCTCAACACTCCAAACTGTGC | 5’-ACTTTAGCTTCGGGTCAATGC |
| *STAT2* | 5’-CCAGCTTTACTCGCACAGC | 5’-AGCCTTGGAATCATCACTCCC |
| *PIK3CD* | 5’-AAGGAGGAGAATCAGAGCGTT | 5’-GAAGAGCGGCTCATACTGGG |

**Table S3.** **List of chemokine signaling pathway related-genes up-regulated in the muscle of DMD subjects.**

| Gene Name | Description | Fold change (vs. Normal) | P value |
| --- | --- | --- | --- |
| *ARRB2* | arrestin beta 2 | 2.440557 | 0.006566 |
| *CCL4* | C-C motif chemokine ligand 4 | 5.781342 | 0.020695 |
| *CCR10* | C-C motif chemokine receptor 10 | 3.808316 | 0.011558 |
| *CXCL11* | C-X-C motif chemokine ligand 11 | 23.35702 | 0.042768 |
| *CXCL12* | C-X-C motif chemokine ligand 12 | 2.130563 | 0.014407 |
| *CXCL16* | C-X-C motif chemokine ligand 16 | 3.920585 | 0.045104 |
| *CXCL9* | C-X-C motif chemokine ligand 9 | 6.853536 | 0.008717 |
| *CXCR3* | C-X-C motif chemokine receptor 3 | 3.303653 | 0.043461 |
| *CXCR4* | C-X-C motif chemokine receptor 4 | 3.017161 | 0.000126 |
| *GNG10* | G protein subunit gamma 10 | 2.481933 | 0.02161 |
| *GNGT2* | G protein subunit gamma transducin 2 | 4.357112 | 0.033149 |
| *GRB2* | growth factor receptor bound protein 2 | 2.155692 | 0.002688 |
| *GRK3* | G protein-coupled receptor kinase 3 | 3.107775 | 0.041661 |
| *PAK1* | p21 (RAC1) activated kinase 1 | 3.396627 | 0.023953 |
| *PIK3CD* | phosphatidylinositol-4,5-bisphosphate 3-kinase catalytic subunit delta | 2.928055 | 0.005099 |
| *PIK3CG* | phosphatidylinositol-4,5-bisphosphate 3-kinase catalytic subunit gamma | 4.267069 | 0.005088 |
| *PLCB2* | phospholipase C beta 2 | 3.409244 | 0.025975 |
| *PRKCB* | protein kinase C beta | 4.184076 | 0.029354 |
| *PTK2B* | protein tyrosine kinase 2 beta | 2.0721 | 0.002487 |
| *PXN* | paxillin | 2.265595 | 0.004177 |
| *RAC2* | Rac family small GTPase 2 | 3.29786 | 0.02502 |
| *SHC1* | SHC adaptor protein 1 | 2.242733 | 0.015109 |
| *STAT2* | signal transducer and activator of transcription 2 | 2.040414 | 0.004174 |
| *WAS* | WASP actin nucleation promoting factor | 3.276829 | 0.017209 |

**Table S4. List of inflammation response related-genes up-regulated in the muscle of DMD subjects.**

| Gene Name | Description | Fold change (vs. Normal) | P value |
| --- | --- | --- | --- |
| *ACER3* | alkaline ceramidase 3 | 2.886769 | 0.03061 |
| *ADAM8* | ADAM metallopeptidase domain 8 | 2.754652 | 0.022536 |
| *ADGRE5* | adhesion G protein-coupled receptor E5 | 3.211791 | 0.003284 |
| *ADORA1* | adenosine A1 receptor | 3.613138 | 0.031868 |
| *AGER* | advanced glycosylation end-product specific receptor | 2.390169 | 0.008142 |
| *AIF1L* | allograft inflammatory factor 1 like | 2.587714 | 0.000252 |
| *BLNK* | B cell linker | 3.045955 | 0.048397 |
| *C4A* | complement C4A (Chido/Rodgers blood group) | 27.74212 | 0.028147 |
| *CAMK1D* | calcium/calmodulin dependent protein kinase ID | 2.31907 | 0.012333 |
| *CAMK4* | calcium/calmodulin dependent protein kinase IV | 2.890538 | 0.049962 |
| *CCL4* | C-C motif chemokine ligand 4 | 5.781342 | 0.020695 |
| *CHST2* | carbohydrate sulfotransferase 2 | 2.039994 | 0.026632 |
| *CIITA* | class II major histocompatibility complex transactivator | 2.779807 | 0.030178 |
| *CSRP3* | cysteine and glycine rich protein 3 | 2.381974 | 0.006845 |
| *CXCL11* | C-X-C motif chemokine ligand 11 | 43.44375 | 0.040815 |
| *CXCL9* | C-X-C motif chemokine ligand 9 | 6.853536 | 0.008717 |
| *CXCR3* | C-X-C motif chemokine receptor 3 | 3.303653 | 0.043461 |
| *CXCR4* | C-X-C motif chemokine receptor 4 | 3.017161 | 0.000126 |
| *ECM1* | extracellular matrix protein 1 | 2.171049 | 0.020118 |
| *FOLR2* | folate receptor beta | 3.764418 | 0.03406 |
| *GBP5* | guanylate binding protein 5 | 3.666607 | 0.010332 |
| *GSDMD* | gasdermin D | 2.017529 | 0.021241 |
| *HYAL3* | hyaluronidase 3 | 3.735343 | 0.048927 |
| *IL17B* | interleukin 17B | 90.71409 | 0.009905 |
| *IL17RA* | interleukin 17 receptor A | 2.716668 | 0.033744 |
| *IL34* | interleukin 34 | 2.391598 | 0.000511 |
| *ITGAL* | integrin subunit alpha L | 3.011155 | 0.017442 |
| *LAT* | linker for activation of T cells | 2.352461 | 0.032001 |
| *LGALS9* | galectin 9 | 2.336315 | 0.011368 |
| *LOXL3* | lysyl oxidase like 3 | 2.36011 | 0.005762 |
| *LTB4R* | leukotriene B4 receptor | 4.385685 | 0.010232 |
| *LY75* | lymphocyte antigen 75 | 3.602679 | 0.043113 |
| *LY86* | lymphocyte antigen 86 | 5.524688 | 0.029693 |
| *LY96* | lymphocyte antigen 96 | 5.044577 | 0.025558 |
| *MEFV* | MEFV innate immunity regulator, pyrin | 10.46322 | 0.008855 |
| *MFHAS1* | multifunctional ROCO family signaling regulator 1 | 2.578558 | 0.008294 |
| *MIF* | macrophage migration inhibitory factor | 2.217607 | 0.017813 |
| *NFKBID* | NFKB inhibitor delta | 2.632563 | 0.023753 |
| *NLRC4* | NLR family CARD domain containing 4 | 2.959637 | 0.007012 |
| *P2RX7* | purinergic receptor P2X 7 | 3.242903 | 0.005552 |
| *PIK3CD* | phosphatidylinositol-4,5-bisphosphate 3-kinase catalytic subunit delta | 2.928055 | 0.005099 |
| *PIK3CG* | phosphatidylinositol-4,5-bisphosphate 3-kinase catalytic subunit gamma | 4.267069 | 0.005088 |
| *PSTPIP1* | proline-serine-threonine phosphatase interacting protein 1 | 2.546209 | 0.018666 |
| *SEMA7A* | semaphorin 7A (JohnMiltonHagen blood group) | 2.664367 | 0.043392 |
| *SIGLEC1* | sialic acid binding Ig like lectin 1 | 4.71366 | 0.021264 |
| *SMAD1* | SMAD family member 1 | 2.148516 | 0.048134 |
| *STK39* | serine/threonine kinase 39 | 2.184378 | 0.041205 |
| *TCIRG1* | T cell immune regulator 1, ATPase H+ transporting V0 subunit a3 | 2.211377 | 0.001785 |
| *TGFB1* | transforming growth factor beta 1 | 4.281608 | 0.000556 |
| *TICAM1* | TIR domain containing adaptor molecule 1 | 2.065763 | 0.001467 |
| *TLR5* | toll like receptor 5 | 3.824666 | 0.002945 |
| *TLR8* | toll like receptor 8 | 6.773462 | 0.006405 |

**Table S5. List of chemokine signaling pathway related-genes up-regulated in the muscle of LGMD subjects.**

| Gene Name | Description | Fold change (vs. Normal) | P value |
| --- | --- | --- | --- |
| *AGER* | advanced glycosylation end-product specific receptor | 2.877262 | 0.001345 |
| *AIF1* | allograft inflammatory factor 1 | 6.048206 | 0.031318 |
| *ALOX15B* | arachidonate 15-lipoxygenase type B | 2.663701 | 0.008334 |
| *CARD9* | caspase recruitment domain family member 9 | 3.343811 | 0.042338 |
| *CD74* | CD74 molecule | 2.143929 | 0.030289 |
| *CSF1R* | colony stimulating factor 1 receptor | 3.768191 | 0.010654 |
| *EGR1* | early growth response 1 | 3.134633 | 0.0219 |
| *F2RL1* | F2R like trypsin receptor 1 | 35.83751 | 0.000843 |
| *FFAR3* | free fatty acid receptor 3 | 4.234753 | 2.85E-05 |
| *HAVCR2* | hepatitis A virus cellular receptor 2 | 4.227541 | 0.028866 |
| *IL4R* | interleukin 4 receptor | 2.592093 | 0.006426 |
| *IL7* | interleukin 7 | 13.84828 | 0.000272 |
| *PYCARD* | PYD and CARD domain containing | 5.168595 | 0.002594 |
| *TLR2* | toll like receptor 2 | 3.201182 | 0.009877 |
| *TLR4* | toll like receptor 4 | 2.442308 | 0.002972 |
| *TLR7* | toll like receptor 7 | 6.554229 | 0.038649 |
| *TNF* | tumor necrosis factor | 6.784575 | 0.004198 |

**Table S6. List of inflammation response related-genes up-regulated in the muscle of DMD subjects.**

| Gene Name | Description | Fold change (vs. Normal) | P value |
| --- | --- | --- | --- |
| *ACKR1* | atypical chemokine receptor 1 | 2.250344 | 0.031376 |
| *ADGRE2* | adhesion G protein-coupled receptor E2 | 5.715355 | 0.004084 |
| *ADGRE5* | adhesion G protein-coupled receptor E5 | 2.39315 | 0.000213 |
| *ADORA1* | adenosine A1 receptor | 5.026114 | 0.001525 |
| *AGER* | advanced glycosylation end-product specific receptor | 2.877262 | 0.001345 |
| *AIF1* | allograft inflammatory factor 1 | 6.048206 | 0.031318 |
| *ANXA1* | annexin A1 | 2.218953 | 0.001981 |
| *BDKRB2* | bradykinin receptor B2 | 4.177424 | 0.015659 |
| *C3* | complement C3 | 7.467413 | 0.019174 |
| *C4A* | complement C4A (Chido/Rodgers blood group) | 11.23599 | 0.000245 |
| *C4B* | complement C4B (Chido/Rodgers blood group) | 9.721141 | 0.000135 |
| *C5AR1* | complement C5a receptor 1 | 10.49107 | 0.013573 |
| *CAMK1D* | calcium/calmodulin dependent protein kinase ID | 2.660262 | 0.02038 |
| *CCL14* | C-C motif chemokine ligand 14 | 3.102495 | 0.008711 |
| *CCL2* | C-C motif chemokine ligand 2 | 9.547329 | 0.007997 |
| *CCL5* | C-C motif chemokine ligand 5 | 3.090347 | 0.007374 |
| *CCR5* | C-C motif chemokine receptor 5 | 3.479264 | 0.038859 |
| *CD180* | CD180 molecule | 11.42491 | 0.016846 |
| *CD44* | CD44 molecule (IN blood group) | 2.576502 | 0.000989 |
| *CHI3L1* | chitinase 3 like 1 | 15.69424 | 0.019391 |
| *CMKLR1* | chemerin chemokine-like receptor 1 | 3.514423 | 0.002619 |
| *CSF1* | colony stimulating factor 1 | 2.063551 | 0.004857 |
| *CSF1R* | colony stimulating factor 1 receptor | 3.768191 | 0.010654 |
| *CXCL10* | C-X-C motif chemokine ligand 10 | 6.698614 | 0.001188 |
| *CXCR3* | C-X-C motif chemokine receptor 3 | 2.494306 | 0.012866 |
| *CXCR4* | C-X-C motif chemokine receptor 4 | 2.325908 | 0.003531 |
| *CYBB* | cytochrome b-245 beta chain | 3.494121 | 0.04286 |
| *DPEP1* | dipeptidase 1 | 17.65018 | 0.020604 |
| *ECM1* | extracellular matrix protein 1 | 3.159291 | 0.001921 |
| *F2RL1* | F2R like trypsin receptor 1 | 35.83751 | 0.000843 |
| *FCGR2B* | Fc gamma receptor IIb | 3.028518 | 0.011642 |
| *FFAR3* | free fatty acid receptor 3 | #DIV/0! | 2.85E-05 |
| *FOXP3* | forkhead box P3 | 4.078391 | 0.04537 |
| *FPR3* | formyl peptide receptor 3 | 4.38259 | 0.002914 |
| *GPR68* | G protein-coupled receptor 68 | 3.10881 | 0.026389 |
| *GSDMD* | gasdermin D | 2.810551 | 0.009769 |
| *HAVCR2* | hepatitis A virus cellular receptor 2 | 4.227541 | 0.028866 |
| *HCK* | HCK proto-oncogene, Src family tyrosine kinase | 5.03652 | 0.024023 |
| *HRH1* | histamine receptor H1 | 2.31942 | 0.033187 |
| *HYAL3* | hyaluronidase 3 | 4.192947 | 0.032048 |
| *IFI16* | interferon gamma inducible protein 16 | 3.20491 | 0.017702 |
| *IGFBP4* | insulin like growth factor binding protein 4 | 3.156168 | 0.001311 |
| *IL17B* | interleukin 17B | 23.23676 | 0.012863 |
| *IL17RA* | interleukin 17 receptor A | 2.302728 | 0.000506 |
| *IL34* | interleukin 34 | 2.302016 | 0.002145 |
| *IRF5* | interferon regulatory factor 5 | 3.45681 | 0.048332 |
| *ITGB2* | integrin subunit beta 2 | 6.183107 | 0.041883 |
| *LACC1* | laccase domain containing 1 | 3.362561 | 0.001379 |
| *LAT2* | linker for activation of T cells family member 2 | 3.618283 | 0.013932 |
| *LGALS9* | galectin 9 | 2.595448 | 0.004535 |
| *LOXL3* | lysyl oxidase like 3 | 2.481482 | 0.000778 |
| *LTB4R* | leukotriene B4 receptor | 2.857346 | 0.017253 |
| *LXN* | latexin | 3.028385 | 0.001377 |
| *LY96* | lymphocyte antigen 96 | 4.687642 | 0.029592 |
| *LYZ* | lysozyme | 5.309665 | 0.024302 |
| *MIF* | macrophage migration inhibitory factor | 2.912222 | 0.013874 |
| *MS4A2* | membrane spanning 4-domains A2 | 3.117451 | 0.038971 |
| *NAIP* | NLR family apoptosis inhibitory protein | 2.424521 | 0.022255 |
| *NFAM1* | NFAT activating protein with ITAM motif 1 | 5.26565 | 0.014683 |
| *NFATC4* | nuclear factor of activated T cells 4 | 3.536576 | 0.044152 |
| *NHLRC4* | NHL repeat containing 4 | 2.182648 | 0.000876 |
| *NKG7* | natural killer cell granule protein 7 | 3.038405 | 0.029368 |
| *NRROS* | negative regulator of reactive oxygen species | 2.807111 | 0.000432 |
| *PIK3CG* | phosphatidylinositol-4,5-bisphosphate 3-kinase catalytic subunit gamma | 2.03157 | 0.031267 |
| *PLD4* | phospholipase D family member 4 | 5.42411 | 0.004931 |
| *PTAFR* | platelet activating factor receptor | 3.813879 | 3.91E-06 |
| *PTGER2* | prostaglandin E receptor 2 | 2.31873 | 0.029601 |
| *PTGER3* | prostaglandin E receptor 3 | 5.955535 | 0.007349 |
| *PTGFR* | prostaglandin F receptor | 6.661007 | 0.045214 |
| *PTGS1* | prostaglandin-endoperoxide synthase 1 | 2.519844 | 0.032931 |
| *PYCARD* | PYD and CARD domain containing | 5.168595 | 0.002594 |
| *SCG2* | secretogranin II | 168.2732 | 0.025067 |
| *SDC1* | syndecan 1 | 6.930915 | 0.042556 |
| *SELE* | selectin E | 5.753292 | 0.049246 |
| *SEMA7A* | semaphorin 7A (JohnMiltonHagen blood group) | 3.45239 | 0.003235 |
| *SERPINA3* | serpin family A member 3 | 32.11643 | 0.023978 |
| *SIGLEC1* | sialic acid binding Ig like lectin 1 | 4.556896 | 0.042413 |
| *SPHK1* | sphingosine kinase 1 | 2.702836 | 0.001872 |
| *STAB1* | stabilin 1 | 6.064911 | 0.021147 |
| *TBXA2R* | thromboxane A2 receptor | 2.515161 | 0.048303 |
| *TCIRG1* | T cell immune regulator 1, ATPase H+ transporting V0 subunit a3 | 2.300439 | 0.000684 |
| *TGFB1* | transforming growth factor beta 1 | 3.83746 | 0.00179 |
| *THBS1* | thrombospondin 1 | 4.426643 | 0.007774 |
| *THEMIS2* | thymocyte selection associated family member 2 | 4.047097 | 0.008022 |
| *TLR2* | toll like receptor 2 | 3.201182 | 0.009877 |
| *TLR4* | toll like receptor 4 | 2.442308 | 0.002972 |
| *TLR7* | toll like receptor 7 | 6.554229 | 0.038649 |
| *TNF* | tumor necrosis factor | 6.784575 | 0.004198 |
| *TNFRSF1A* | TNF receptor superfamily member 1A | 2.000941 | 0.016313 |
| *TNFRSF1B* | TNF receptor superfamily member 1B | 2.238171 | 0.001033 |
| *TNFRSF4* | TNF receptor superfamily member 4 | 4.877381 | 0.020625 |
| *TPST1* | tyrosylprotein sulfotransferase 1 | 2.508662 | 0.001506 |
| *VNN1* | vanin 1 | 7.467063 | 0.034296 |

**Table S7. Human subject characteristics**

| **Name (Code)** | **Date of birth** | **Height (cm)** | **Age** | **Body weights (kg)** | **Date of treatment** | **Date of sample collection** | **Disease** |
| --- | --- | --- | --- | --- | --- | --- | --- |
| A1 | 02/02/2009 | 124.5 | 10.0 | 29.2 | —— | 15/02/2019 | LGMD2D |
| A2 | 18/10/2011 | 120 | 7.6 | 29.1 | —— | 6/05/2019 | DMD |
| A3 | 01/09/2012 | 111 | 5.9 | 19.8 | —— | 9/07/2018 | DMD |
| A4 | 03/05/2013 | 117 | 8.0 | 26.7 | 1/01/2020 | 15/02/2021 | DMD |
| A5 | 03/02/2011 | 121 | 8.4 | 24.9 | —— | 19/07/2019 | LGMD2B |
| A6 | 07/08/2011 | 119 | 7.7 | 23.9 | —— | 18/03/2019 | DMD |
| A7 | 12/06/2013 | 107 | 4.4 | 18.9 | —— | 18/05/2018 | DMD |
| A8 | 12/04/2010 | 110.5 | 8.4 | 28.5 | —— | 7/05/2018 | LGMD2C |
| A9 | 04/06/2014 | 116 | 4.1 | 19.4 | —— | 28/05/2018 | DMD |
| A10 | 26/08/2009 | 119 | 8.8 | 29.5 | —— | 8/06/2018 | LGMD2B |
| A11 | 16/05/2012 | 111 | 7.6 | 22.6 | 15/06/2018 | 16/12/2019 | DMD |
| A12 | 31/12/2012 | 123 | 7.5 | 24 | 15/06/2019 | 15/06/2020 | DMD |
| A13 | 10/07/2010 | 132.4 | 8.1 | 28.3 | —— | 31/08/2018 | LGMD2C |
| A14 | 10/06/2013 | 106 | 5.9 | 17.9 | —— | 6/09/2019 | LGMD2D |
| A15 | 06/09/2008 | 124.5 | 11.1 | 24.2 | —— | 20/07/2019 | LGMD2C |
| A16 | 18/11/2014 | 97 | 5.0 | 15.4 | —— | 14/11/2019 | DMD |
| A17 | 10/03/2012 | 120.7 | 8.0 | 35.4 | —— | 27/03/2020 | DMD |
| A18 | 04/03/2015 | 98.2 | 4.1 | 16.5 | 22/04/2018 | 16/04/2019 | DMD |
| A19 | 21/08/2013 | 113 | 6.3 | 17.3 | —— | 29/11/2019 | LGMD2D |
| A20 | 08/05/2011 | 125 | 7.6 | 24.5 | —— | 27/11/2018 | DMD |
| A21 | 19/05/2012 | 118 | 6.5 | 21 | —— | 3/12/2018 | LGMD2B |
| A22 | 07/03/2014 | 95.5 | 4.5 | 15.3 | 1/05/2017 | 18/01/2019 | DMD |
| A23 | 14/09/2014 | 110 | 4.8 | 20 | —— | 5/07/2019 | DMD |
| A24 | 01/01/2012 | 115 | 7.2 | 19.8 | —— | 2/03/2019 | LGMD2C |
| A25 | 02/05/2014 | 100 | 4.9 | 16 | —— | 22/12/2018 | DMD |
| A26 | 07/05/2013 | 106 | 6.2 | 25 | 8/10/2019 | 27/09/2020 | DMD |
| A27 | 21/05/2011 | 122 | 7.8 | 26 | 12/04/2019 | 1/03/2021 | DMD |
| A28 | 05/05/2014/ | 84 | 4.8 | 13.3 | —— | 29/03/2019 | DMD |
| A29 | 03/02/2013 | 105 | 6.1 | 20 | 29/03/2019 | 29/03/2020 | DMD |
| A30 | 10/01/2011 | 123.1 | 7.5 | 25 | 28/06/2019 | 25/03/2020 | DMD |
| A31 | 10/07/2011 | 116.5 | 7.7 | 20.4 | 5/07/2019 | 5/07/2020 | DMD |
| A32 | 07/06/2012 | 115 | 7.0 | 18.5 | 5/07/2019 | 5/07/2020 | DMD |
| A33 | 28/01/2012 | 114 | 7.4 | 20 | 5/07/2019 | 5/07/2020 | DMD |
| A34 | 17/02/2015 | 105 | 4.5 | 18 | 5/08/2019 | 5/08/2020 | DMD |
| A35 | 22/02/2012 | 106 | 7.5 | 21.3 | —— | 5/08/2019 | DMD |
| A36 | 11/03/2011 | 115.6 | 7.7 | 20.1 | —— | 13/08/2019 | DMD |
| A37 | 14/10/2014 | 108 | 4.8 | 16.8 | 12/08/2018 | 16/08/2019 | DMD |
| A38 | 20/04/2015 | 127 | 4.4 | 19 | 16/09/2019 | 16/09/2020 | DMD |
| A39 | 01/08/2012 | 120 | 7.2 | 23 | 30/09/2019 | 30/09/2020 | DMD |
| A40 | 12/02/2012 | 111 | 6.9 | 21 | 18/10/2019 | 21/10/2020 | DMD |
| A41 | 11/12/2013 | 105.5 | 6.0 | 18.6 | 10/05/2020 | 28/10/2021 | DMD |
| A42 | 06/04/2015 | 114 | 7.2 | 16 | 27/10/2021 | 20/09/2022 | DMD |
| A43 | 05/11/2014 | 93 | 4.2 | 12.5 | —— | 7/07/2018 | DMD |
| A44 | 27/05/2012 | 108.5 | 6.1 | 19 | —— | 12/07/2018 | DMD |
| A45 | 11/07/2010 | 115.7 | 7.7 | 21.5 | —— | 13/07/2018 | DMD |
| A46 | 04/11/204 | 162 | 14.3 | 70 | —— | 16/07/2018 | Controls |
| A47 | 08/07/2010 | 110 | 7.9 | 20 | —— | 16/07/2018 | Controls |
| A48 | 04/05/2009 | 125.6 | 9.2 | 23.5 | —— | 27/06/2018 | Controls |
| A49 | 01/12/2009 | 117.6 | 8.5 | 18.6 | —— | 20/07/2018 | Controls |
| A50 | 20/08/2009 | 118.2 | 8.9 | 27 | —— | 20/07/2018 | Controls |
| A51 | 31/08/2010 | 124.3 | 8.3 | 23.5 | —— | 20/07/2018 | Controls |
| A52 | 27/05/2013 | 110 | 9.2 | 17 | —— | 27/07/2022 | Controls |
| A53 | 27/10/2011 | 119 | 6.8 | 21 | —— | 27/07/2018 | Controls |
| A54 | 30/05/2009 | 119.9 | 9.2 | 22.5 | —— | 30/07/2018 | Controls |
| A55 | 21/02/2012 | 110.3 | 6.4 | 18.5 | —— | 30/07/2018 | Controls |
| A56 | 08/09/2008 | 125.2 | 10.0 | 18.7 | —— | 24/08/2018 | Controls |
| A57 | 24/07/2008 | 145 | 10.1 | 44 | —— | 20/08/2018 | Controls |
| A58 | 16/11/2014 | 109.7 | 5.8 | 18.8 | —— | 4/09/2020 | Controls |
| A59 | 29/10/2015 | 109.5 | 4.9 | 17 | —— | 4/09/2020 | Controls |
| A60 | 16/03/2016 | 109.7 | 5.9 | 21 | —— | 8/02/2022 | Controls |
| A61 | 17/03/2012 | 124 | 8.8 | 22.6 | —— | 14/12/2020 | Controls |
